# Recent Advances in MEMS-Based Microthrusters

**DOI:** 10.3390/mi10120818

**Published:** 2019-11-26

**Authors:** Bendong Liu, Xinrui Li, Jiahui Yang, Guohua Gao

**Affiliations:** 1 College of Mechanical Engineering and Applied Electronics Technology, Beijing University of Technology, Beijing 100124, China; Lixinrui_yy@163.com (X.L.); klrs80@sohu.com (J.Y.); gaoguohua@bjut.edu.cn (G.G.); 2 Electrical and Mechanical College, Beijing Vocational College of Agriculture, Beijing 102208, China

**Keywords:** microthrusters, micropropulsion, microspacecraft, microfabrication, MEMS technology

## Abstract

With the development of micro/nano satellites and formation flying, more advanced spatial propulsion technology is required. In this paper, a review of microthrusters developments that based on micro electromechanical systems (MEMS) technology adopted in microthrusters is summarized. The microthrusters in previous research are classified and summarized according to the types of propellants and the working principles they utilized. The structure and the performance including the thrust, the impulse and the specific impulse of various microthrusters are compared. In addition, the advantages and the disadvantages of these microthrusters presented in the paper are discussed.

## 1. Introduction

Micro/nano satellites have been gaining more attention in recent years not only due to reducing the costs of launch and mission, but also increasing reliability of spacecraft [1]. With the development of microsatellite technology and the expansion of its application field, the attitude control, drag compensation, station keeping, and orbit adjustment functions of microspacecraft have put forward more urgent needs for micropropulsion systems. Thrust levels of a few milli-Newton or less and impulse bits as little as 10^−6^ Ns may be required for attitude control of a microspacecraft [2]. Such micropropulsion systems may be especially important for microspacecraft attitude control due to their achievable small size and mass [3]. Micro electromechanical systems (MEMS) technology plays a very important role in the development of micropropulsion systems. The fabrication technology of MEMS is conventionally defined as silicon-based batch processing techniques. However, the acronym MEMS is used almost universally to refer to the devices produced by the microfabrication process [4], such as bulk and surface micromachining as well as high-aspect-ratio micromachining (HARM) selectively remove parts of the silicon or add structural layers to form the mechanical and electromechanical components. By using MEMS technology, some small devices up to the level of micron can be fabricated with very precise shape [5]. In addition, the ability to fabricate the lightweight and very small-volume devices also becomes increasingly important for microspacecraft.

The microfabrication technology of MEMS has been successfully applied in the field of micropropulsion for micro/nano satellites. Many kinds of micropropulsion systems based on MEMS technology have been investigated in the past years. As shown in Table 1, the main microthrusters researched by several groups can be roughly categorized according to the driving method they adopted. In this review, we intend to classify solid propellant microthrusters (SPM) [6] and liquid monopropellant microthruster [7,8] as chemical fuel propellant microthrusters. Additionally, the SPM is categorized into vertical structure design and planar structure design according to their structure and the liquid monopropellant microthruster is classified into two types of catalytic decomposition method: Spark ignition and catalytic ignition. Other microthrusters mainly depended on electric driving including the vaporizing liquid microthruster (VLM) [9,10], plasma microthruster [11,12,13], electrospray microthruster [14,15,16], and colloid microthruster [17,18,19].The free molecule micro–resistojet (FMMR) is also an electrothermal micropropulsion system, which is designed and used for the on-orbit maneuvers of nanosatellites [20,21]. Furthermore, cold gas microthruster (CGM) [22,23,24,25] works by compression and release of high-pressure gas.

All these types of microthruster are in the process of continuous development, which also represent the potential of MEMS technology. In this paper is a brief review of the current status of development on the various microthrusters that can be manufactured using MEMS technologies including structure design, innovation, and performance testing of various types of microthruster. Additionally, the form of table is adopted to compare most of the present research of various kinds of microthrusters. However, some kinds of microthrusters have less research than others that we could obtain from current studies, such as CGM, plasma microthruster, electrospray microthruster, colloid microthruster, and FMMR. Therefore, some microthrusters with representative structure are briefly introduced.

## 2. Chemical Fuel Propellant Microthruster

The chemical fuel propellant microthrusters mainly include the SPM and the liquid monopropellant microthruster. The SPM produces huge energy of up to a few thousand Newtons of thrust by energetic fuel combustion, which requires a microigniter. The SPM has the following major advantages: (1) The simple structure of SPM can reduce the system complexity and increase the system miniaturization, which leads to reliable functioning and assembly; (2) SPM has the character of no moving parts, which can eliminate the problem of friction; (3) there is no liquid fuel in the SPM, therefore the possibility of propellant leakage is very low compared with others microthrusters that use gas or liquid propellant. Moreover, the propellant of SPM is stable in time; (4) the SPM also has an ability to combine compact structure and size with a small amount of power. However, the major disadvantage of the SPM is one-shot use (it can only work one time). Thus, it is necessary to adopt the design form of propulsion array to solve the problem of multiple ignitions due to its one-off ignition consumption [26]. The liquid monopropellant microthruster mainly depends on catalytic liquid chemical propellant decomposition as the energy source. The main advantage of liquid monopropellant microthruster is that it can offer a relative great range of total impulse. Moreover, thrust level and the impulse of the liquid monopropellant microthruster can be controlled by adjusting the duration or the flow rate of liquid chemical propellant.

### 2.1. Solid Propellant Microthruster (SPM)

**Figure 1:**
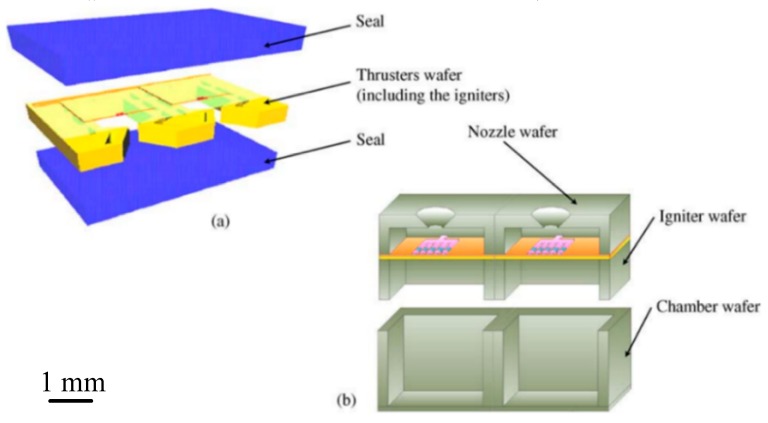
Structural concept diagram of (**a**) the planar structure design and (**b**) the vertical structure design. [27] Copyright © 2006, Elsevier B.V. With permission of Elsevier.

Generally, a SPM contains a combustion chamber, a microigniter, a membrane, and a micronozzle. The principle of the SPM operation is that a solid energetic material is used as propellant and stored in the combustion chamber, and then, the solid propellant is heated up by a microigniter until temperature of its ignition is reached. Some gases are produced after combustion and the membrane of SPM is broken by the gases. Then, the gases are accelerated through a micronozzle and a thrust is produced. Ordinarily, silicon and glass (Pyrex, photosensitive glass) were mostly used as the materials for a solid propellant microthruster due to the harsh working condition of the microthruster. According to the structure design of the SPM, it can be classified into two kinds of structures: Vertical structure design and planar structure design, as shown in Figure 1. For the vertical structure design, the different structure units of microthruster were fabricated on different silicon wafers and assembled finally to form the microthruster devices, which required appropriate bonding technology. The SPM with planar structure contains a nozzle, a microigniter, and a chamber in the same wafer, which can be completed in one micromachining step. In addition, the vertical structure design is more suitable for the design of SPM arrays because of its character of suitable for large-scale integration. Thus, the research on the vertical structure design has become a hot spot. This section mainly introduces the structure and performance of the SPM, the special design of different microigniters, the adoption of the materials, and various bonding methods.

#### 2.1.1. SPM with Vertical Structure

**Figure 2:**
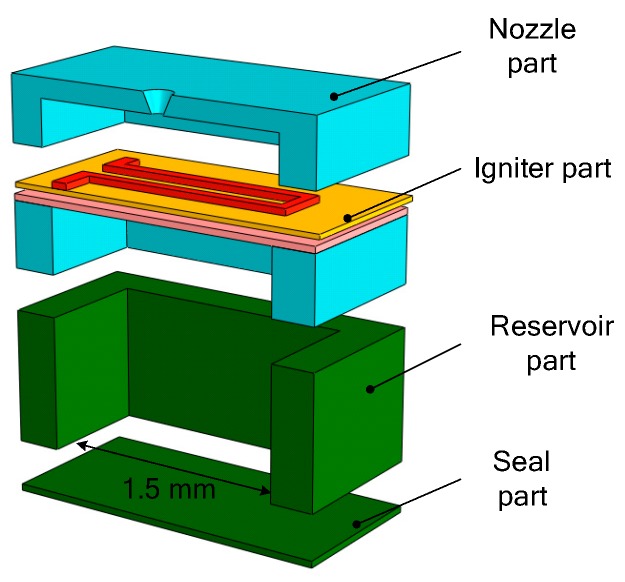
Schematic structure of a single solid propellant microthruster (SPM) consisted in a stack of four wafers with a top igniter. Figure modified from Ref. [31].

**Figure 3:**
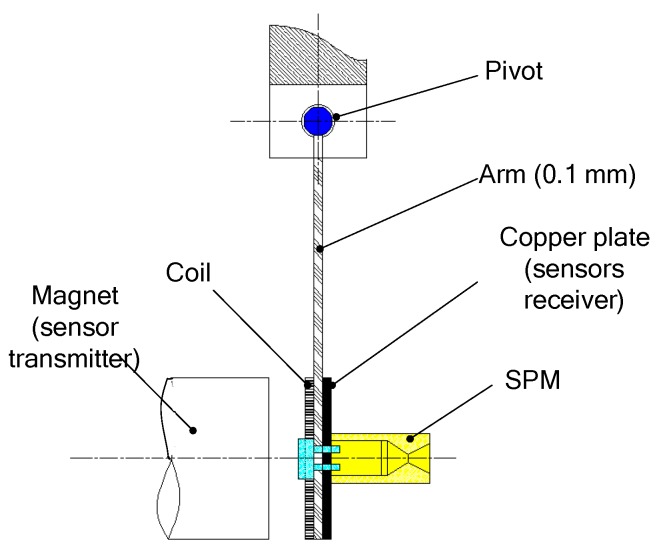
Schematic view of the thrust measurement stand. Figure modified from Ref. [31].

Different research teams have started the research of the SPM based on MEMS technology [28,29,30] since the end of the 20th century. Generally, a SPM was fabricated in a silicon wafer and the thrust generated ranged from a few μN to a few mN. B. Larangot et al. [31] reported a MEMS-based SPM array in silicon wafer with vertical structure, which consisted of a stack of four wafers: A nozzle layer, an igniter layer, a chamber layer, and a seal part, as shown in Figure 2. The chamber was fabricated using deep reactive ion etching (DRIE) of silicon. The microigniter is a polysilicon microheater thermally fabricated by a series of micromachining on the top of the igniter layer, which is isolated on a SiO_2_/SiN*_x_* dielectric membrane. The adhesive bonding of thermal epoxy (H70E, Polytec) and a UV sensitive polymer (NOA 88, Norland) was chosen for the assembling of the microthruster. A thrust stand consisting of a thin and rigid arm (100 µm thick) rotating freely around a pivot was built as shown in Figure 3. When the thrust force is applied, the pendulum rotates around the pivot axis. A displacement sensor feeds a voltage to the electronic control. Measurement of the equilibrium force equal to the thrust is obtained by measuring the electric current. In the process of testing, the rotating friction and environmental interference are inevitable, which will reduce the measurement accuracy.

**Figure 4:**
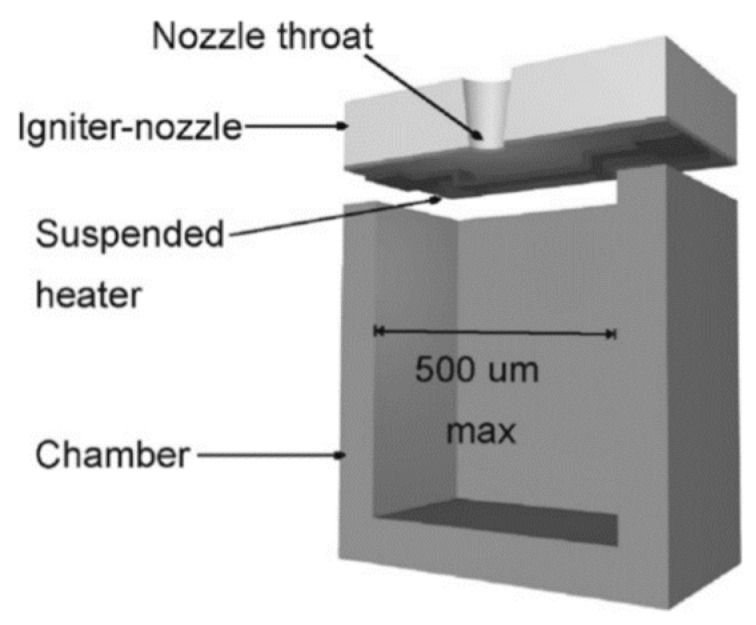
Schematic structure of a SPM composed of two parts. [32] Copyright © 2006 Elsevier B.V. With permission of Elsevier.

In order to simplify the processes of fabrication and the assembling, Briand et al. [32] presented an optimal SPM with a surface micromachined polysilicon microheater and the number of layers composing the microthruster was reduced from 4 to 2, as shown in Figure 4. The suspended microheaters were thermally isolated from the silicon substrate though an air gap. The microheaters were made with P-doped polysilicon resistors and could be integrated around the micronozzle throat directly. In addition, the thermal losses through the substrate also could be minimized by suspending the microheaters in this way.

**Figure 5:**
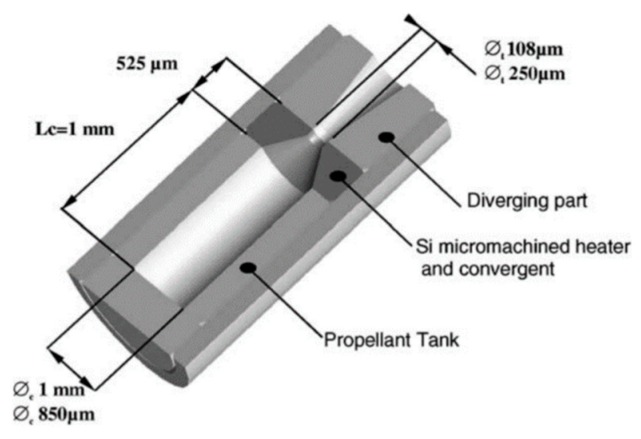
Schematic view of a single SPM consisted of three silicon parts. [33] Copyright © 2002 Elsevier Science B.V. With permission of Elsevier.

Rossi et al. [33] reported a SPM that consisted of three silicon parts: A silicon micromachined igniter, a propellant tank, and a diverging part wafer, as shown in Figure 5. Each part of the SPM was firstly filled with the solid propellant and assembled with epoxy mixture glue (EPO TEK H70E) cured at 60 ℃ during l5 h to form the SPM complete structure. The microigniter was realized by a patterned polysilicon resistor on a very thin dielectric membrane. The thrust force of the SPM ranged from 1 mN to a few mN.

**Figure 6:**
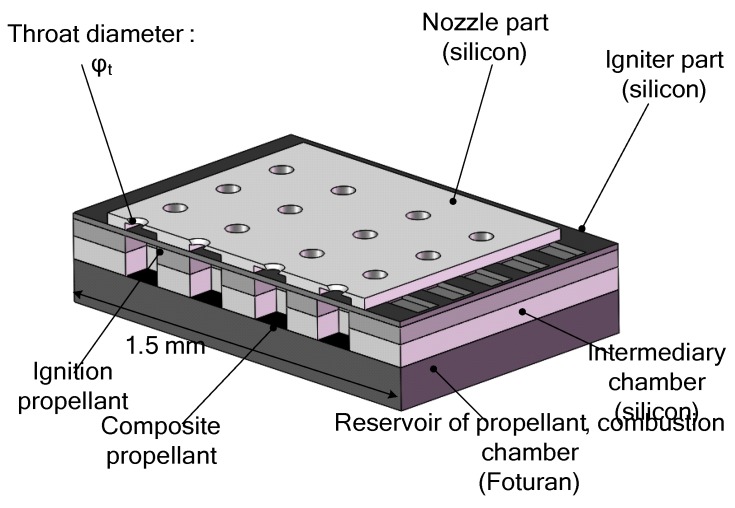
Schematic of an array of 16 SPM designed by Rossi et al. Figure modified from Ref. [34].

**Figure 7:**
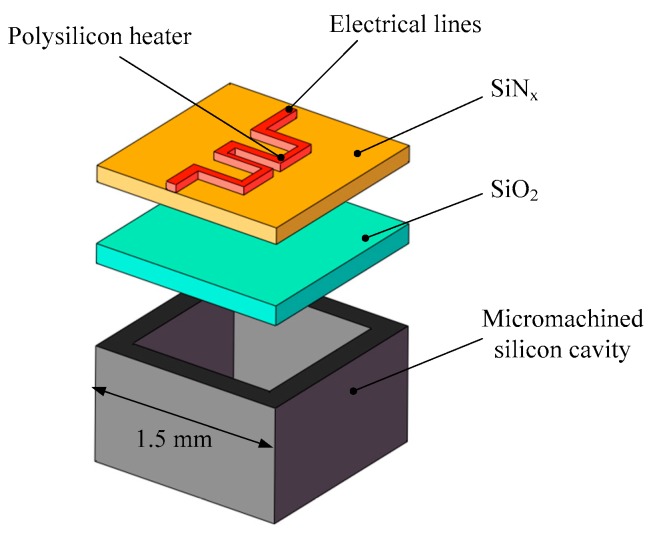
Schematic view of a single silicon micromachined igniter. Figure modified from Ref. [34].

**Figure 8:**
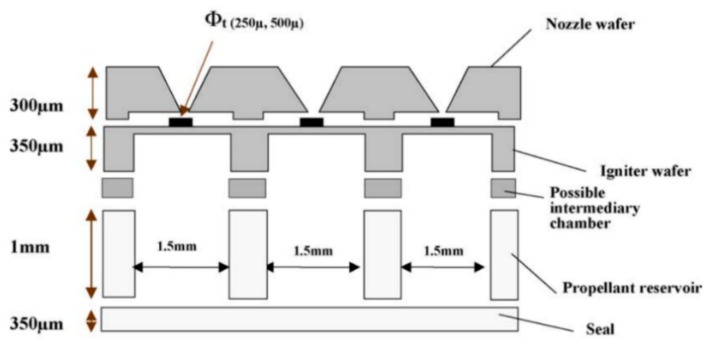
Schematic view of an array of 100 SPM structure designed by Rossi et al. [27] Copyright © 2006, Elsevier B.V. With permission of Elsevier.

Subsequently, due to the fact that a single SPM has the disadvantage of one-off shot, more SPM arrays have been developed with the continuous progress of MEMS technology. The SPM arrays of 16 and 100 solid propellant microthrusters were presented by Rossi et al. [27,34]. The structure of an array of 16 SPM is shown in Figure 6. There are four main parts, including a silicon micromachined igniter, a propellant reservoir in Foturan, which also is used as the combustion chamber, and a micronozzle wafer made in silicon. The composition of a single silicon micromachined igniter is shown in Figure 7. The ignition success rates of the GAP (glycidyle szide polymer) and ZPP (zirconium perchlorate potassium) material are 75% and 100%, respectively. The SPM generated thrusts reached 22 mN with ZPP combustion and the throat nozzle dimension of 500 μm. The main factor affecting the ignition success rate is that debris is created when the membrane is broken during the combustion [35]. The thin dielectric membrane rupturing too early will cause the failure of ignition. The structure and dimensions of a SPM array of 100 designed by Rossi et al. [34] are shown in Figure 8. The first silicon layer contains the micronozzles and with *Φ*250 μm throats. The microigniters and addressing elements are fabricated in the second silicon layer. The third layer comes with the propellant reservoirs fabricated in a photoetchable glass or a silicon wafer and the bottom Pyrex layer is the seal layer of the device. In the assembling and bonding process, a Pyrex seal wafer was glued to the chamber wafer with an UV sensitive polymer (NOA 88, Norland) before receiving the propellant and the nozzle part was bonded to the igniter part by a thermal epoxy (H70E, Polytec). The thrust measurement system was also adopted the method proposed in the study of Larangot et al. [31] The combustion of the GAP was in the chamber and the thrust generated ranged from 0.3 mN to 2.3 mN.

**Figure 9:**
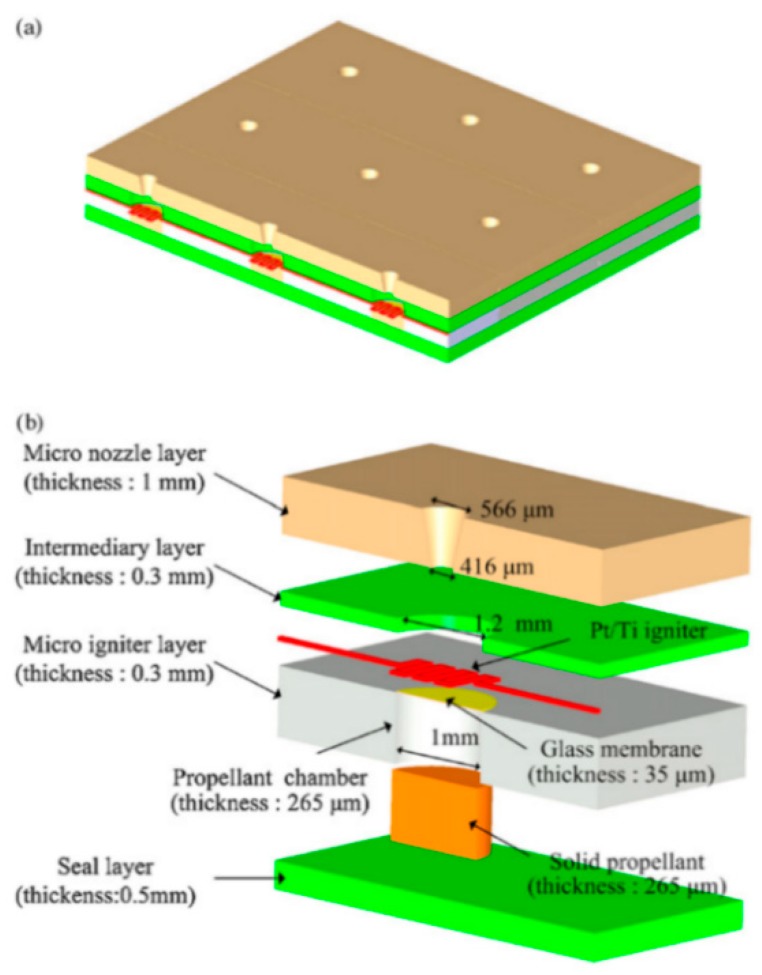
Schematic of the array of 3 × 3 SPM structure designed by Lee et al. [36] Copyright © 2009 Elsevier B.V. With permission of Elsevier.

The selection of the structural materials in the design of SPM is also an important aspect that needs to be considered because an SPM operates in mechanically and thermally harsh conditions. Several studies currently have tried to combine silicon and glass (Pyrex, photosensitive glass) together. Lee et al. [36] proposed that a photosensitive glass wafer was adopted to fabricate a microthruster according to its characters of low thermal conductivity, low fabrication cost, and high integration. A schematic of a 3 × 3 SPM array is illustrated in Figure 9. After the propellant filling process, UV curable glue, cured by UV exposure, was adopted to complete the finally bonding. Then, the SPM array was assembled into a printed circuit board (PCB) using gold wire bonding. Platinum (Pt) was used in the fabrication of the heating resistor due to it has the advantages of high stability at high temperature, resistance to oxidation, and corrosion [37]. The stability of the microigniter was improved by using a glass membrane with the average thickness of 35 μm compared with the thin SiO_2_/SiN*_x_* dielectric membrane. A piezoelectric sensor (Kistler, 9205) was used to measure the thrust and total impulse. The sensor produced an electric charge and the charge meter (Kistler, 5015A) converted the electric charge into voltage. After testing, the averages of the maximum thrust and the total impulse were 3619 mN and 0.381 mNs, respectively. The average specific impulse was 62.3 s.

**Figure 10:**
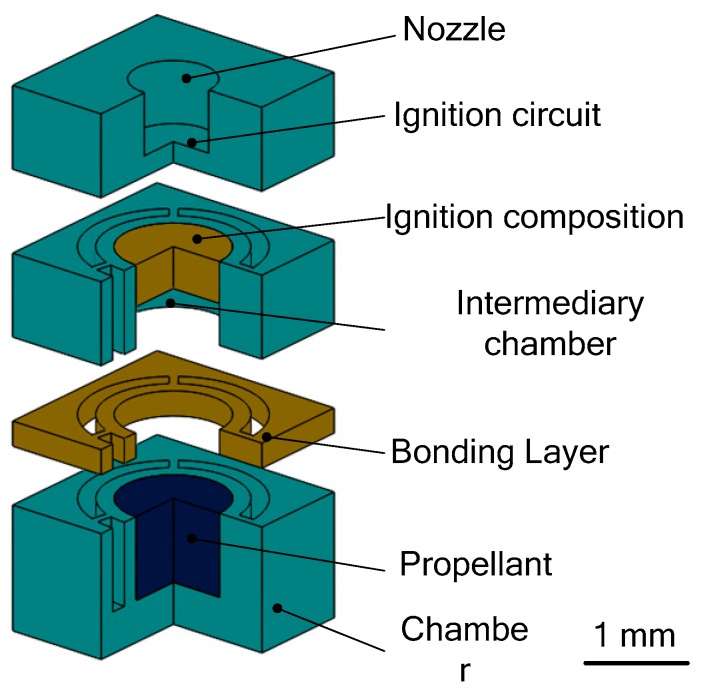
Schematic of the SPM with a top ignition designed by Ru et al. Figure modified from Ref. [40].

**Figure 11:**
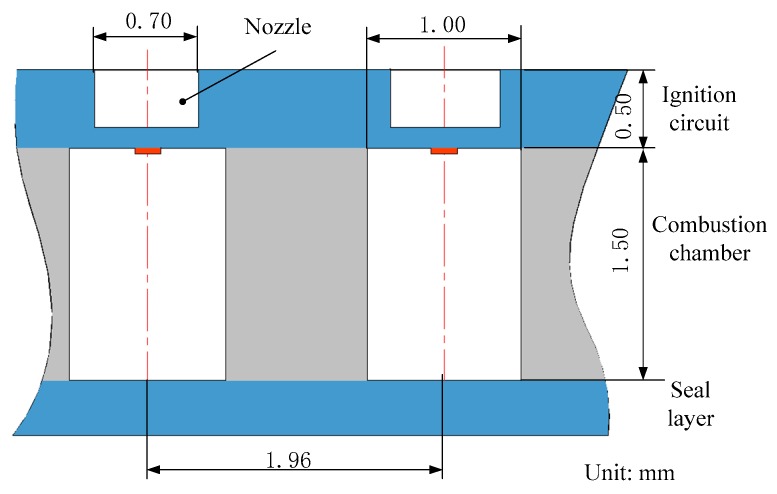
Structure and dimensions of a 10 × 10 SPM array units loaded with nano-Al/CuO mixture. Figure modified from Ref. [41].

**Figure 12:**
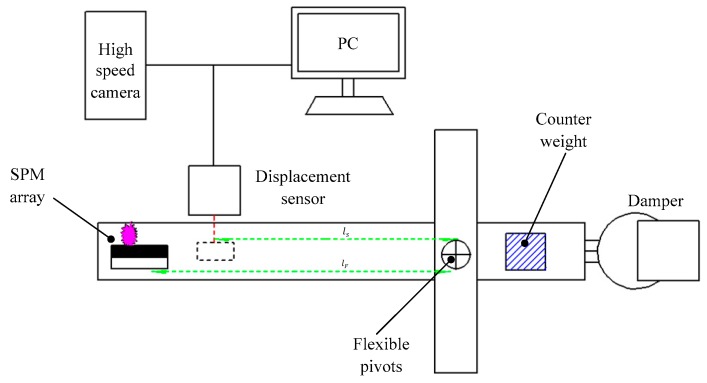
Schematic view of the impulse testing system. Figure modified from Ref. [41].

In addition, it is also essential for the vertical structure SPM to select an appropriate bonding technology. With the developing of the MEMS, various bonding technologies have been researched to meet some special requirements of different products in MEMS field [38]. The main bonding technologies include silicon-to-silicon fusion bonding, anodic bonding, room temperature silicon-to-silicon bonding [39], adhesive bonding, solder bonding, and eutectic bonding [40]. Generally, adhesive bonding was adopted for the assembling process due to contamination of the surface after filling propellant. Ru et al. [40] developed a vertical structure SPM that adopted a bonding technique of low-temperature eutectic alloy for the assembling of the SPM array by considering the safety of the charge filled in the chambers and the operational reliability. The SPM structure with top-side igniter is shown in Figure 10. The intermediate layer bonding process can tolerate some particles, contaminations, or structure on the surface of the substrates in lower temperature. The eutectic alloy bonding process involved the submerging the chamber array into the molten alloy, which was composed of Bi-Pb-Sn-Cd-In (117# Indalloy, Indium Corporation) and the melting point is only 47 °C. After that, Ru et al. [41] fabricated a 10 × 10 SPM array prototype, which was loaded with nano-Al/CuO mixture. One-component epoxy adhesive (HHCK 6917-14) was used to assemble the array under 150 °C at 4.7 MPa pressure for 2 min. In order to improve the thrust performance of SPM, nanothermites mixed with different nitrocellulose (NC) contents were adopted as propellant in the microthrusters, which was also adopted in the research of Staley et al. [42,43] and Apperson et al. [44,45]. The structure and dimensions of one SPM unit are shown in Figure 11; a straight micronozzle was finally used because a Laval nozzle is too complex in terms of fabrication processing [41]. The diaphragm membrane that supported the igniter was located between the nozzle and the ignition circuit in order to protect the propellant from the rugged space environment. The impulse testing system consists of a displacement sensor, damper, flexible pivots, counterweight, and torsional pendulum, as shown in Figure 12. The SPM array was mounted at the end of torsional pendulum. When the SPM worked, the position of pendulum was tracked by the displacement sensor. The test showed that the specific impulse and total impulse with binder-free nano-Al/CuO reached 10.2 s and 155.9 μN·s, respectively. The average thrust ranged from 479 mN to 645 mN. However, it is difficult to obtain the initial balance of this measurement system as it is disturbed by the environment.

**Figure 13:**
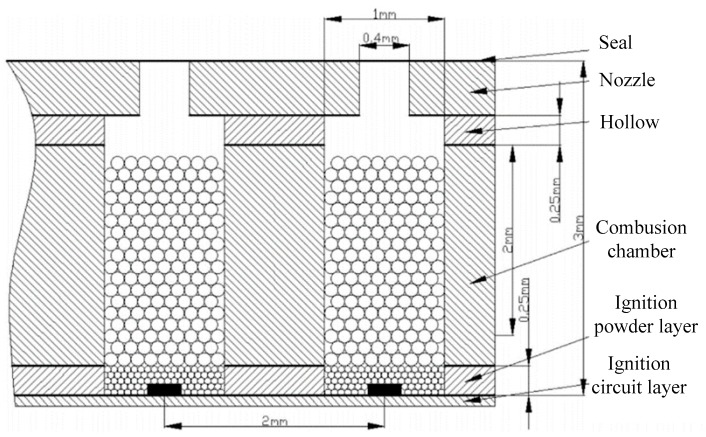
Structure diagram of a single SPM of a 10 × 10 array designed by Liu et al. [46] Copyright © 2015 Elsevier B.V. With permission of Elsevier.

Due to the fact that epoxy resin has a better heat insulation property and low thermal conductivity, it was adopted to fabricate microchambers in order to avoid undesired ignition of neighboring units in the study of Liu et al. [46]. SPM array prototypes of 10 × 10 and 100 × 100 scale were both developed, and the propellant was hydroxyl-terminated polybutadiene/ammonium perchlorate (HTPB/AP) with a diameter that ranged from 100 μm to 200 μm. The microthruster mainly consists of five parts, including ignition circuit layer, bottom ignition powder layer, propellant layer, cavity layer, and straight micronozzle layer, as shown in Figure 13. Epoxy resin glue was also used to achieve all layers together. The thrust was directly measured by a piezoelectric force sensor (208C01). Tests showed that the lowest power of 0.72 W can realize the ignition. Then, the total impulse of the 10 × 10 array ranged from 0.058 mN·s to 0.147 mN·s.

#### 2.1.2. SPM with Planar Structure

The main characteristics of the SPM with planar structure are that the parts of the microthruster are all integrated in the same wafer, reducing the fabrication steps and the bonding techniques. Moreover, the shape of the micronozzle can be easily adjusted according to different demand, compared with the SPM with vertical structure.

**Figure 14:**
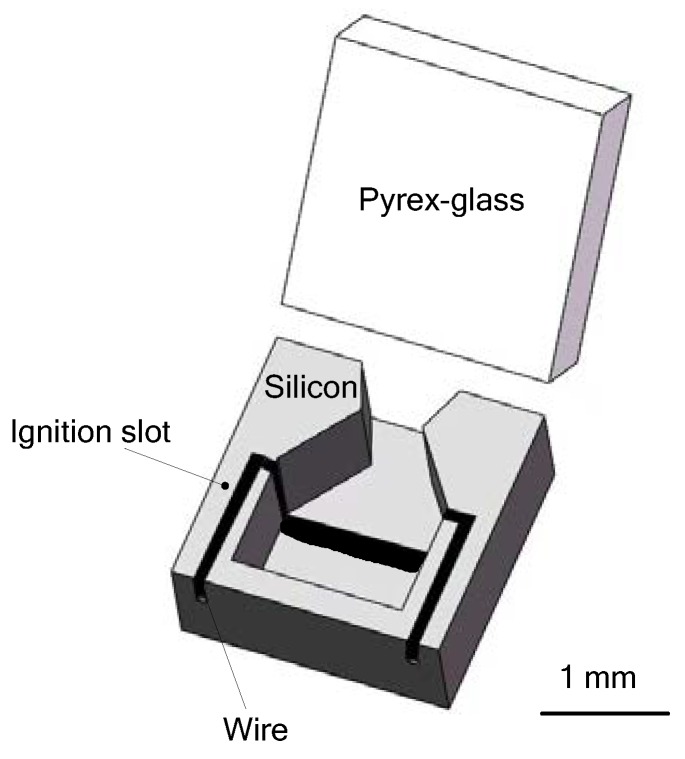
Schematic view of a single SPM with a special wire adopted as the igniter. Figure modified from Ref. [47].

Zhang et al. [47] proposed a SPM with planar structure composed only two wafer layers. A combustion chamber, an ignition slot, and a convergent-divergent nozzle were fabricated in a silicon wafer. A specific glass layer with a same dimension was bonded together with the silicon wafer utilizing the anodic bonding at 400 °C and a voltage of 1100 V to realize a complete microthruster device. HTPB/AP/AL (Adding aluminum) was chosen as the solid propellant for the microthruster and a special ignition wire was installed in the ignition slot to ignite the propellant. Figure 14 shows the schematic view of such a single microthruster. The design with planar structure has several advantages of higher efficiency in fabrication, more flexibility in design, and better bonding quality to ensure the reliability of the product. However, it is not optimal for integration and batch fabrication that a wire was adopted as the igniter. Moreover, its microigniter does not allow low power consumption.

**Figure 15:**
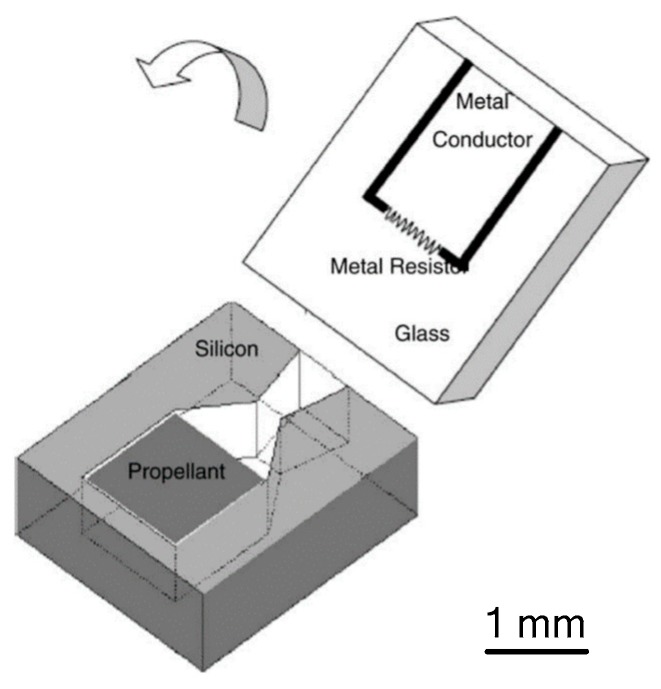
Schematic view of a single SPM with a new igniter of Ti and Au, respectively, as the resistor and the conductor. [48] Copyright © 2005 Elsevier B.V. With permission of Elsevier.

**Figure 16:**
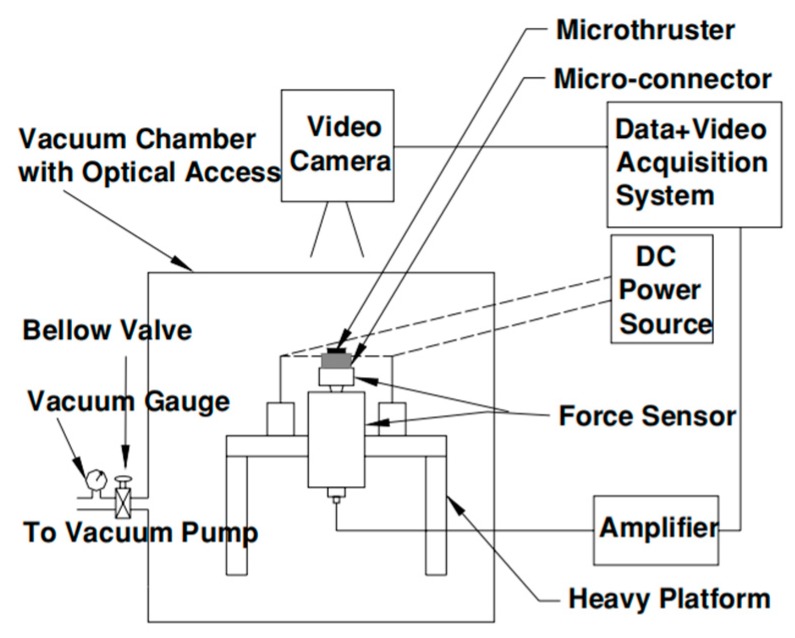
Schematic view of the thrust vacuum testing system in the study of Zhang et al. [48] Copyright © 2005 Elsevier B.V. With permission of Elsevier.

In 2005, Zhang et al. [48] demonstrated a SPM with planar structure using an anodic bonding method and integrated it with an Au/Ti igniter, which is more suitable for batch fabrication and high-level integration. The schematic of a proposed single microthruster is shown in Figure 15. A metal igniter was fabricated on a sodium-rich glass substrate with a lift-off processing. A combustion chamber and a convergent–divergent nozzle were fabricated on a silicon wafer using a series of microfabrication technologies. The new igniter had a resistor made of Ti and conductor made of Au instead of the wire igniter, which improved the ignition efficiency the reliability and integration. Gunpowder-based solid propellant was adopted for the experimental testing. The schematic view of the thrust vacuum testing system is shown in Figure 16. A Kistler quartz force sensor was adopted to measure the thrust force produced by the low-temperature co-fired ceramic (LTCC) microthruster. The measurement environment in the vacuum state can reduce the influence of the environment. However, the products generated from the combustion of propellants cannot be cleaned up in time, which will also interfere with the test results. The results showed that the thrust peak value ranged from 0.05 N to 0.34 N and total impulse ranging from 2.11 × 10^−5^ Ns to 1.15 × 10^−4^ Ns were produced at sea level.

**Figure 17:**
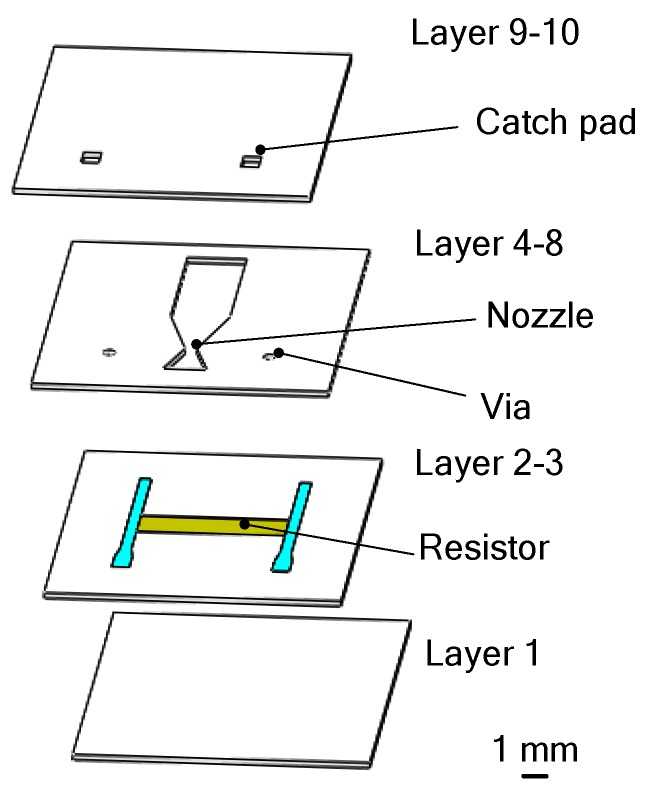
Schematic view of the low-temperature co-fired ceramic (LTCC) SPM designed by Zhang et al. Figure modified from Ref. [49].

Except silicon-based SPM, LTCC technology has been developed for the fabrication of the SPM [49] with planar structure in 2005. As shown in Figure 17, a LTCC microthruster was fabricated with the lamination of several green tapes and each layer was individually processed. After the processing of all layers was completed, they were all stacked in a laminating fixture plate and then sealed in a vacuum bag and placed in an isostatic laminator system at 800 psi and 70 °C for 10 min. This was followed by sintering of the stack in a programmable forced-draft furnace. The microthruster mainly consists of a combustion chamber, a convergent–divergent nozzle, and a resistor embedded inside the combustion chamber. The resistor was connected to the catch pads on the top of the microthruster through electrical vias. The propellant ignition was triggered by electrical power applied to the ignition resistor through the catch pads and interconnections. The microthrusters were also tested employing gunpowder-based solid propellant. Using the same testing system in the study of Zhang et al. [48], the results showed that the total impulse ranged from 3.81 × 10^−5^ N·s to 1.27 × 10^−4^ N·s and the specific impulse ranging from 5.55 s to 14.41 s was produced at sea level.

**Figure 18:**
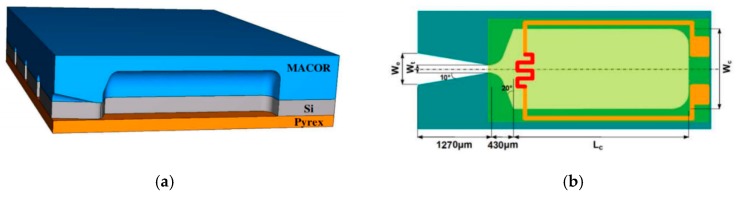
Schematic of a SPM with planar structure composed of three layers: Ceramic, Si, and Pyrex glass layers; (**a**) the structure of 7 × 1 SPM array. (**b**) The top view of a single microthruster. ©2015 IOP Publishing Ltd. Reprinted, with permission, from [50].

**Figure 19:**
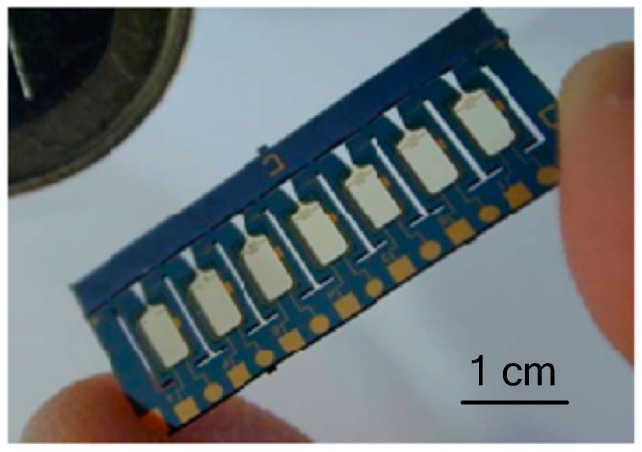
Photograph of 7 × 1 micromachined silicon chip for silicon array designed by Chaalane et al. ©2015 IOP Publishing Ltd. Reprinted, with permission, from [50].

Chaalane et al. [50] developed a 7 × 1 SPM array with planar structure. The schematic of the SPM structure is shown in Figure 18. The microthruster was composed of three layers, which are one silicon wafer layer with a top-side igniter and a micromachined nozzle, a ceramic (Macor) layer with the chamber extension was glued on the top side of the silicon layer, and a glass (Pyrex) layer glued on the silicon bottom side using H70-E epoxy glue. A fabricated silicon chip prepared for the assembly step is shown in Figure 19. The polysilicon resistor was fabricated on a low-stress SiO_2_/SiN*_x_* thin membrane, which was used as the microigniter. DB + *x*%BP was employed as propellant for experimental tests. A thrust stand reported by Orieux [51] was adopted in the thrust force measurement. The disturbances present in the curve during data acquisition were caused by the material of measurement and the environment air. Thrust ranging from 0.1 mN to 3.5 mN was obtained with a throat width of 100 µm.

#### 2.1.3. Summary of SPM and SPM Arrays

Besides the above studies, Lewis et al. [26] in 2000, You et al. [52] in 2005, Rossi et al. [53] in 2005, Sathiyanathan et al. [54] in 2011, and Shen et al. [55] in 2014 also developed different SPM arrays. The vertical design has advantages of high integration level and convenient expansion. However, the bonding between layers may be very difficult when the chip is filled with energetic material, which makes the bonding process unsuitable at high temperature. Thus, the development of low-temperature bonding process at more than 180 °C and less than 500 °C is very important to improve the safety and reliability of the SPM devices.

Additionally, the ignition mode of the SPM includes bottom ignition and top ignition. For bottom ignition, the unreacted propellants and combustion products will rush out together when the pressure in the combustion chamber exceeds the mechanical strength of the thin membrane, which will reduce the utilization rate of propellants and cause thrust loss. For top ignition, the combustion is more adequate. However, in the process of fabricating or assembling, the ignition circuit with the top ignition will be easily failed because of the insufficient strength of the thin membrane. In conclusion, the SPM has been researched extensively by various groups in the past years [26,56]. The performance of SPM and SPM arrays studies by different researchers are summarized in Table 2. Abbreviations of propellants that need to be explained include the primary explosive lead styphnate (LTNR), glycidyle azide polymer/ammonium perchlorate/ zirconium (GAP/AP/Zr), and propellant composed of a double-base and black-powder (DB + *x*%BP).

### 2.2. Liquid Monopropellant Microthruster

The liquid monopropellant microthruster uses some liquid chemical fuel, also known as a liquid chemical fuel microthruster, such as hydrazine, hydroxylammonium nitrate, and hydrogen peroxide(H_2_O_2_), as a propellant. Although hydrazine has the advantage of high energy, it also has toxic exhaust, which is not conducive to the development of microthrusters. The H_2_O_2_ is considered a “green” fuel because it decomposes into a benign mixture, which consists of water vapor and oxygen, when it is exposed to a catalyst [57]. The liquid monopropellant microthruster is classified into spark ignition and catalytic ignition according to its ignition process. Spark ignition is realized by a pair of electrodes when the voltage of the electrodes reaches the breakdown voltage of the material in between [58]. Catalytic ignition is achieved with liquid chemical fuel, such as H_2_O_2_, decomposed by means of some catalysts. The operating principle of liquid monopropellant microthrusters is that a liquid propellant is injected into a reaction chamber where it decomposes into hot gas when in contact with a catalyst material or a pair of electrodes and then accelerated through a micronozzle to generate thrust. Ceramics are usually adopted in the fabrication of liquid monopropellant microthrusters because of it has the characters of high-temperature tolerance and good chemical resistance. Additionally, compared with silicon, the low thermal conductivity of ceramics also means it is usually adopted. The main advantage of a liquid chemical fuel microthruster is that its operational life proportional to the size of chamber and a wider range of thrust and impulse can be obtained by adjusting the flow rate and duration of the liquid propellants. However, the problem of unstable and easy decomposition of propellants also should be given more attention.

#### 2.2.1. Liquid Monopropellant Microthruster with Spark Ignition

Spark ignition is realized with a pair of metal electrodes integrated inside the microthruster in order to directly contact with the liquid propellant and the energy can be deposited directly into the propellant liquid.

**Figure 20:**
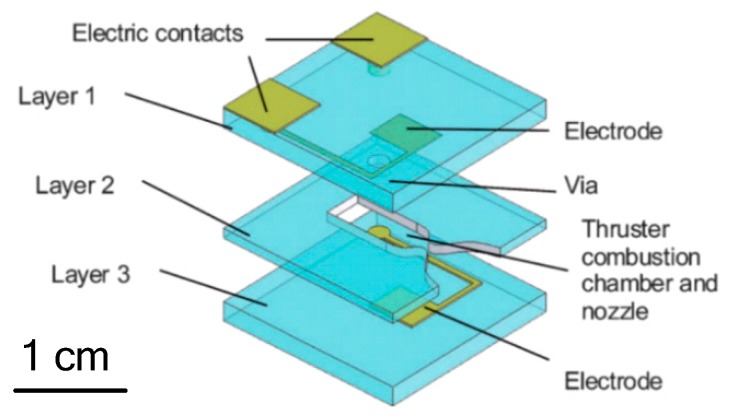
Schematic view of the LTCC electrolytic microthruster designed by Wu et al. ©2009 The Royal Society of Chemistry. Reprinted, with permission, from [58].

**Figure 21:**
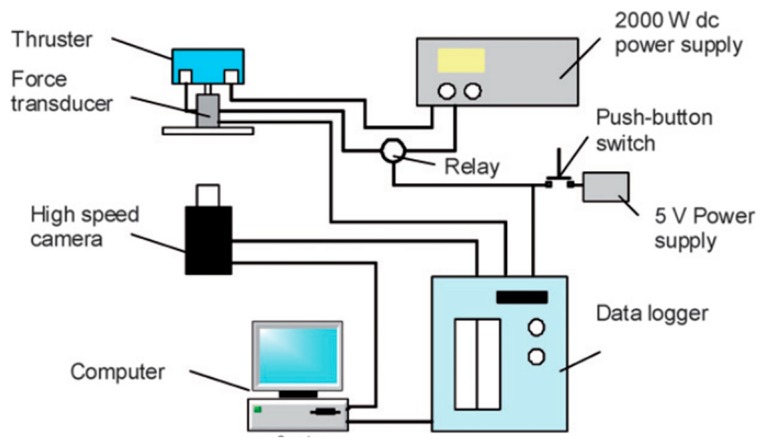
Schematic view of the thrust measurement system for LTCC electrolytic microthruster designed by Wu et al. ©2009 The Royal Society of Chemistry. Reprinted, with permission, from [58].

Wu et al. [58] designed a planar liquid monopropellant microthruster, which was fabricated with LTCC technology. The structure of the LTCC electrolytic microthruster is comprised of three major layers, as shown in Figure 20. The microthruster combustion chamber and micronozzle were fabricated on Layer 2 using micropunches in alumina (Al_2_O_3_)-based ceramics. A pair of metal electrodes was designed on the surfaces facing the microthruster chamber on the Layer 1 and Layer 3, respectively. The honey/water mixture was brushed on the surfaces of major layers and all layers were stacked together. The whole LTCC tape stack was placed in a vacuum bag and then placed into a programmable oven for co-firing. The firing process comprised of two steps: (1) The oven temperature was ramped slowly to 500 °C at a rate of 1.5 °C/min from room temperature and held for 1 h for the burn-out of organic fluid for lamination and the binder in the LTCC tapes, (2) being ramped up again to the sintering temperature of 850 °C at a rate of 5 °C/min. The oven was kept at 850 °C for 30 min for the sintering of the ceramic and then naturally cooled to room temperature. The electrodes were designed on both the entire top and bottom surfaces of the chamber in order to better contact the liquid propellant. Figure 21 shows the thrust measurement system for a LTCC electrolytic microthruster. A microforce transducer (PCI, U209C11) with a resolution of 0.1 mN was adopted in the system. Thrust levels ranging from approximately 100 mN to 200 mN have been obtained by using a DC voltage potential ranged from 20 V to 60 V to decompose HAN (hydroxyl ammonium nitrate). However, there are some cracking in the combustor wall because of the thermal stressing under rapid ignition conditions. Vissotski [59] proposed that reducing cross-sectional area and decreasing propellant flowrate can realize a controllable level for thermal stresses within the LTCC.

**Figure 22:**
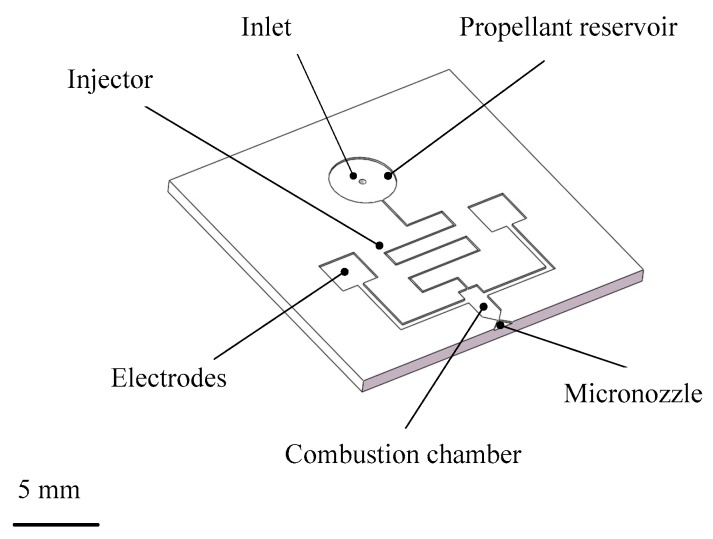
Structure design of the zirconia micro electromechanical systems (MEMS)-based microthruster designed by Cheah et al. Figure modified from Ref. [60].

Cheah et al. [60] fabricated a planar zirconia microthruster with fabrication process of gel casting and PDMS soft molds. The structure of the microthruster is shown in Figure 22. The gel-casting technique was selected to provide a rapid consolidation of the ceramic suspension. Pre-sintered YSZ (yttria-stabilized zirconia) micro-parts were infiltrated with the pre-ceramic resin to seal the pores and were subsequently converted to SiOC at a relatively low temperature of 900 °C. The propellant can be decomposed into gases when electrical energy is supplied, which is realized by two conductive paths connected to both sides of the chamber. The structure of microthruster consists of only two layers, which has the advantages of simple structure and convenient manufacture.

#### 2.2.2. Liquid Monopropellant Microthruster with Catalytic Ignition

For a liquid monopropellant microthruster with catalytic ignition, silver (Ag) and platinum (Pt) are usually used as catalytic metals decompose the H_2_O_2_ propellant. In addition, transition metal oxides also have been used as catalysts to decompose the H_2_O_2_ propellant [61,62] due to their significant activities, in which pure and mixed manganese oxides are a major choice for H_2_O_2_ decomposition [63,64]. Hitt et al. [65] presented a H_2_O_2_ microthruster in 2001, in which the silver catalytic pillars were adopted in a combustion chamber. However, due to the fact that the H_2_O_2_ microthruster will significantly reduce the activity of the catalyst if it is be operated long term at low temperatures, especially for silver catalysts, the phenomenon of incomplete decomposition and poor performance of thrust efficiency at the micronozzle exit were obtained in experimental results. In order to shorten the delay time of ignition and improve the defect of incomplete decomposition, Kuan et al. [66] proposed a H_2_O_2_ microthruster with a microheater to pretreat the catalyst bed, which can produce a thrust level of more than 100 mN. The thrust measurement of H_2_O_2_ microthruster was carried out at a condition of 420 K used as a preheating temperature. Test results showed that 92 wt.% H_2_O_2_ at a flow rate of 0.18 g/s with the catalyst of Ag could generate a thrust of 182 mN and a specific impulse bit of 101 s under atmospheric pressure.

**Figure 23:**
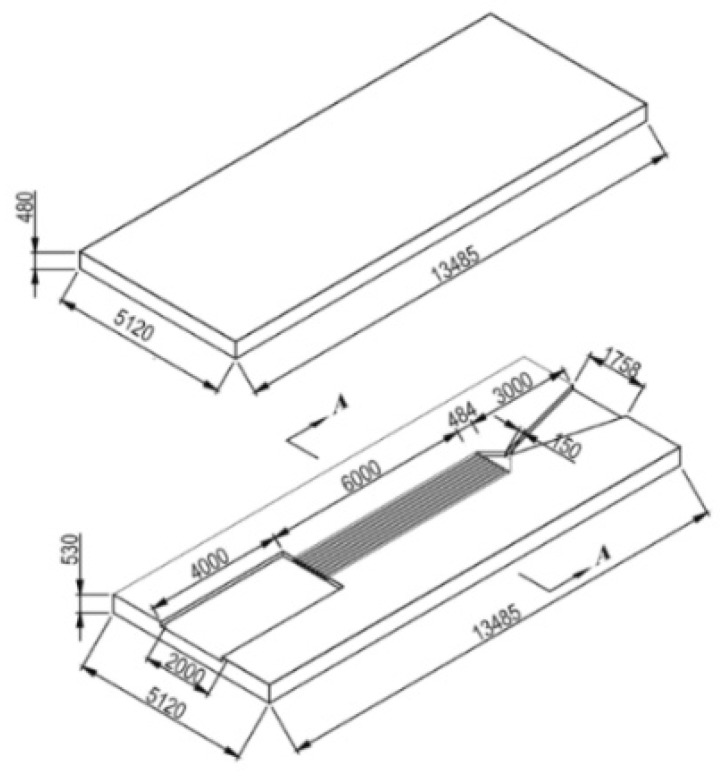
Structure view of the microthruster chip with nine parallel microchannels (unit: μm). [67] Copyright ©2010 Elsevier Ltd. With permission of Elsevier.

**Figure 24:**
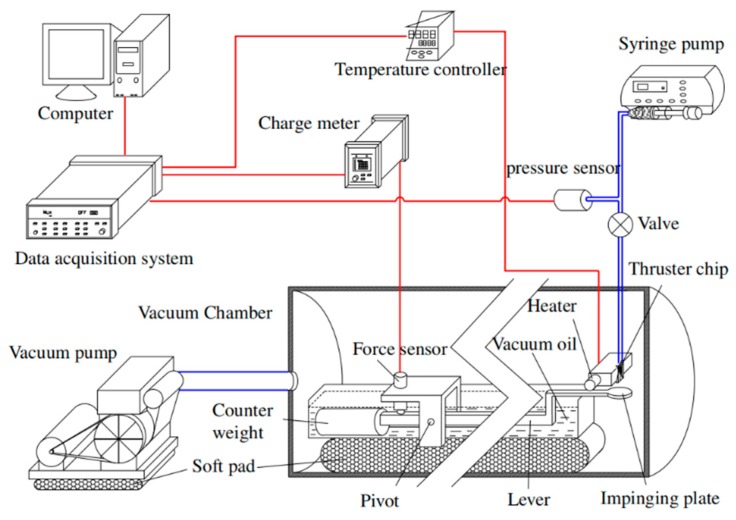
Structure view of the thrust measurement system. [67] Copyright ©2010 Elsevier Ltd. With permission of Elsevier.

Cen and Xu [67] reported a H_2_O_2_ propellant microthruster chip fabricated by etching on silicon. As shown in Figure 23, the microthrust consists of a Pyrex glass layer and a silicon substrate with a rectangular inlet, nine parallel microchannels, and a convergent-divergent nozzle. The metal Pt was adopted as the catalytic metal, which was sputtered on the inner surface of the nine parallel microchannels in order to improve the efficiency of heating. The Pyrex glass layer and the silicon layer were bonded together to complete the microthruster device with anode bonding process. Moreover, the surface of microthruster was smeared with heat conduction cream and then the microthruster was stickled to a piece of copper with a heater inside. The structure view of the thrust measurement system is shown in Figure 24. The microthruster spurted gas at the impinging plate and the resulting impinging force was enlarged by a lever. Then, the enlarged force was sensed by a piezoelectric sensor set on the other side of the lever. The whole system was placed on a bed of sponge to reduce disturbance from the ground such as a noise occurring come from someone running nearby. The temperature of the copper was measured by a thermal couple. However, the results showed that due to the thermal capacity of the microthruster being too small, controlling the temperature stably became very difficult. The test showed that a maximum thrust was 6.5 mN with a mass flow rate of 500 mg/min and the specific impulse ranged from 65 s to 105 s at different temperatures and flow rates.

**Figure 25:**
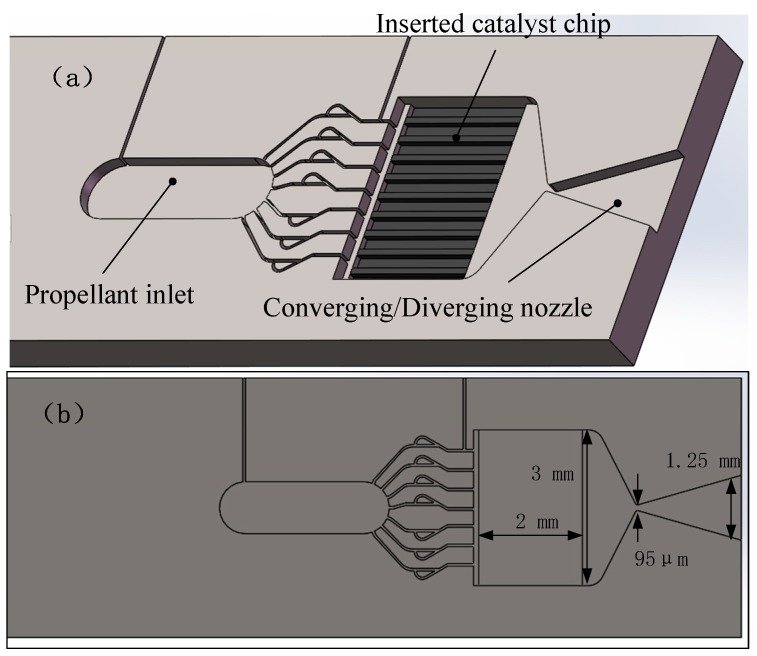
A 3D structure of microthruster chip with Pt thin film as resistance temperature detector (RTD). Figure modified from Ref. [68].

In order to better control of the internal temperature of the microthruster chamber, Miyakawa et al. [68] fabricated a microthruster, which integrated Pt thin film in microthruster as a resistance temperature detector (RTD). One layer of microthruster consists of an inlet, a catalyst chamber, and a converging/diverging nozzle, which was fabricated using the process of DRIE on a silicon wafer. The other layer was sputtered Pt thin film as RTD and had a microheater. Finally, the catalyst chip was inserted into the recess of reaction chamber and glued manually by using ceramic adhesive. Figure 25 shows the 3D structures of the microthruster. The tested heating power of microthruster was up to 20 W and the inside temperature distribution was also measured by integrated RTDs, which showed a much better thermal performance.

**Figure 26:**
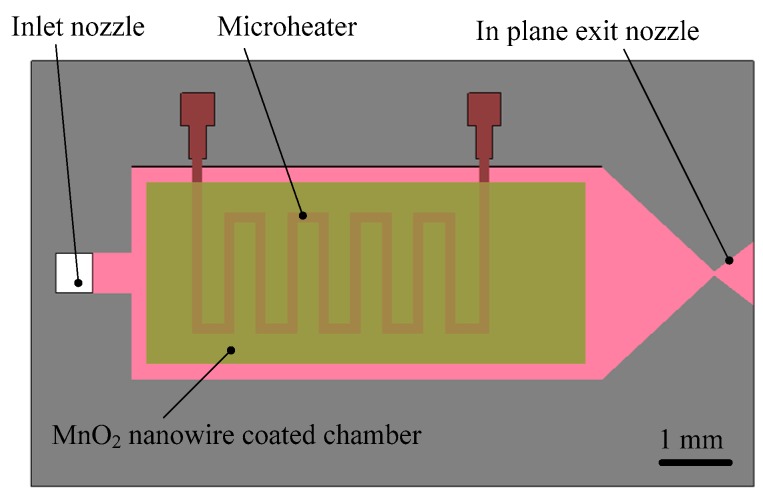
Structure design of MnO_2_ nanowire-embedded microthruster. Figure modified from Ref. [69].

Kundu et al. [69] presented a chemically synthesized MnO_2_ nanowire catalyst-embedded H_2_O_2_ monopropellant microthruster and the microheater realized by boron-diffused meanderline resistor was fabricated to preheat the reaction chamber. The structure design of the proposed microthruster with MnO_2_ nanowire embedded is shown in Figure 26. The device is composed of an inlet nozzle, a microchannel, a MnO_2_ nanowire-embedded reaction chamber, a planar exit nozzle, and a microheater, which are all integrated in a silicon wafer. It was also confirmed that the nanowires are firmly attached to the chamber surface by self-adherence technique (Van-der walls force) after the alcohol was completely vaporized and surface was dried up. A Pyrex glass with no micromachining parts was adopted as the top layer that bonded with the micromachined silicon wafer to realize the complete microthruster device. A bonding process using polymer adhesive glue has been employed instead of standard wafer bonding technique for the realization of the microthruster. A high-sensitivity laboratory balance (DV215CD, OHAUS Discovery Semi-micro Dual Range Balance) of resolution 0.1 μN (0.01 mg) connected to a data acquisition facility in Lab view software using RS232 port with average data sampling rate of 8.6 Hz was used for thrust measurement. The thrust force ranging from 0.3 mN to 1.1 mN could be produced with 50 wt.% H_2_O_2_ at flow rates of 0.20–1.25 mg/s and the specific impulse ranged from 80 s to 180 s with consumption of electrical power of 2.0–2.2 W to preheat at 423 K for 20 s.

**Figure 27:**
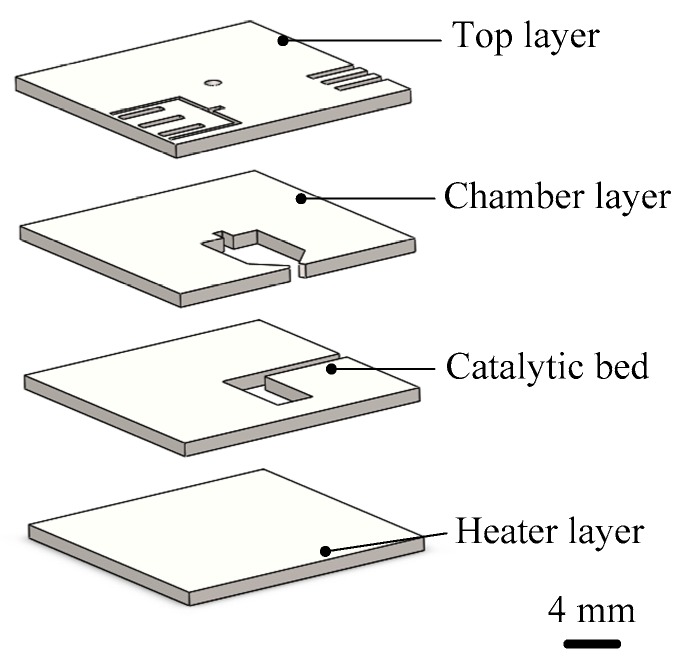
Exploded schematic diagram of alumina microthruster. Figure modified from Ref. [71].

Khaji et al. [70,71] designed a liquid monopropellant alumina microthruster with an integrated heater. The microthruster is comprised of four ceramic tapes stacked on each other as shown in Figure 27. The structure mainly consists of a bottom layer with a heater and temperature sensor, a catalytic bed layer, a reaction chamber layer including a chamber, an inlet channel, and a nozzle, and a top layer with leads and an additional temperature sensor. Before characterization, copper wires (200 µm in diameter) were connected to the electrical contacts of the devices using conductive epoxy (CW2400, Chemtronics, USA). The microthruster demonstrated complete evaporation at a power above 3.7 W and a propellant flow rate of 50 µL/min with 30 wt.% H_2_O_2_. Under these conditions, a thrust and specific impulse of 0.96 mN and 106 s were obtained by calculations, respectively.

**Figure 28:**
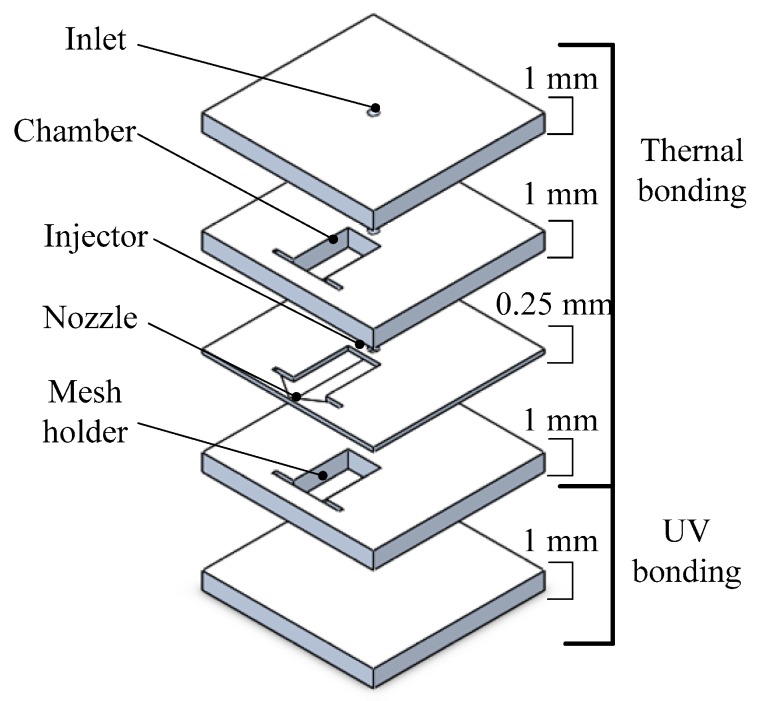
Schematic view of microthruster components. Figure modified from Ref. [72].

**Figure 29:**
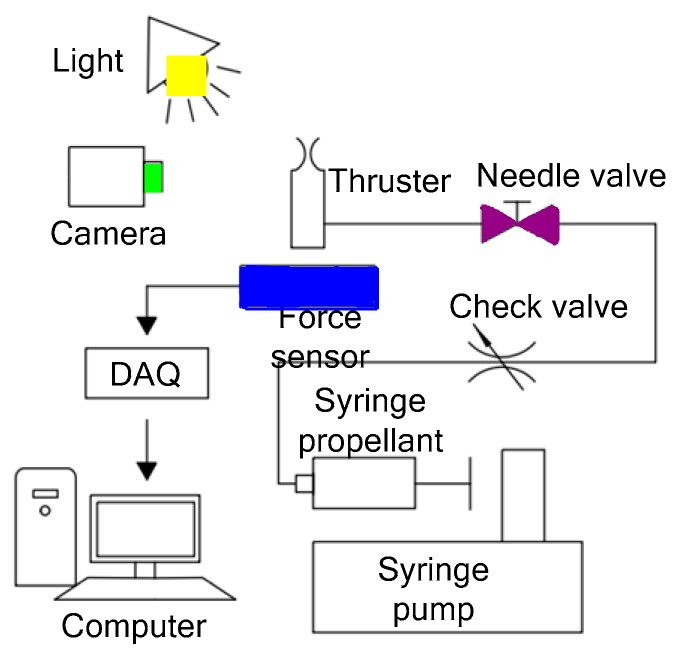
Schematic view of thrust measurement system for performance test. Figure modified from Ref. [72].

It is important to offset the excessive heat loss caused by the small scale of the microthruster, which can be offset by using high-energy content as a propellant. Thus, Huh and Kwon [72] developed a method to enhance the energy content of the propellant by blending 90 wt.% H_2_O_2_ with ethanol at an oxidizer. The structure of the microthruster consisted of five glass layers and each one was patterned with the processing of lithography and wet etching individually, as shown in Figure 28. Four layers of thruster were joined by thermal bonding conducted at 500 °C for 12 h under 2100 Pa in a furnace and the last layer by a UV bonding technique. The UV bonding was achieved with liquid adhesive (Optical Adhesives and Coatings, OP24 produced by DYMAX) on a spin coater rotating at 4000 rpm for 50 s. Then, 1.5 min of exposure under 310 nm UV light completed the bonding process. The active material of Pt, which was separately prepared on the gamma alumina pellets with wet impregnation, was adopted as a decomposition catalyst and inserted into the reaction chamber. The experimental setup for the performance test is shown in Figure 29. A force sensor (Kistler, model 9205), propellant feed system with a motor driven syringe pump, and imaging devices were installed in the system. Finally, the transient voltage signal that was proportional to the thrust was transferred to a computer and the value of thrust could be obtained. The measured thrusts reached approximately 30 mN with 1.7 mL/min of the blended H_2_O_2_. However, the measured thrust was approximately 24 mN when the flow rate of the pure H_2_O_2_ was 1.7 mL/min.

**Figure 30:**
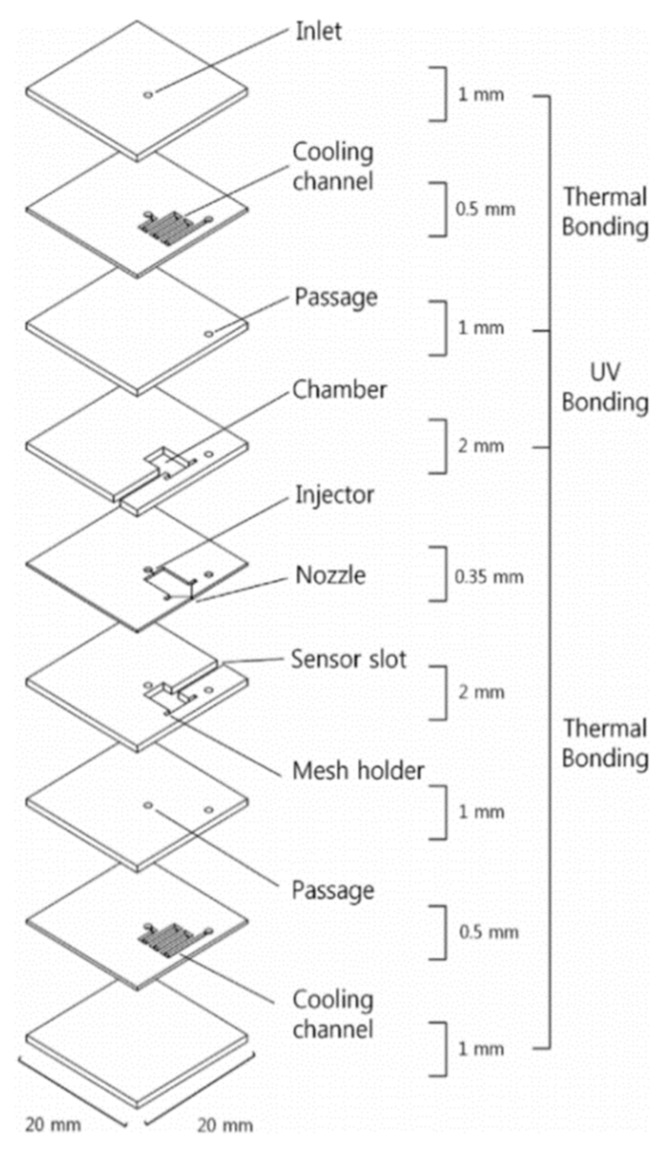
Schematic view of the microthruster with micro-cooling channels. [73] Copyright © 2017, Elsevier B.V. With permission of Elsevier.

Although glass has been used as a structural material in microthruster fabrication to reduce the heat loss, it is also a challenge to solve the frangibility of the glass. Thus, Huh et al. [73] subsequently presented a microthruster with built-in regenerative micro-cooling channels, which consists of nine photosensitive glass layers. Figure 30 shows a schematic view of the microthruster with the cooling channels on each wafer. Before integration of the layers, a Pt/Al_2_O_3_ catalyst was inserted into the chamber for propellant decomposition. The method of UV bonding and performance test system adopted also were same as previous research [72]. The 90 wt% H_2_O_2_ was both used as propellant and served as the working fluid for regenerative cooling. The test results showed that the microthruster generated thrust of 48 mN and specific impulse of 70.4 s.

#### 2.2.3. Summary of Liquid Monopropellant Microthruster

Table 3 presents the data for comparison extracted from the references of liquid monopropellant microthruster developed by different researchers. For liquid monopropellant microthruster with spark ignition, it is necessary but complicated to connect the electrodes nearby the reaction chamber with the wire to provide larger electric energy. On the other hand, for liquid monopropellant microthruster with catalytic ignition, there is a problem that the thrust generation and the efficiency of propellant decomposition may be insufficient when a catalyst coated on the inner surface of the microthruster chamber. Additionally, the decomposing of H_2_O_2_ depends on using various catalysts, which has a small demand for electricity. Although the propellants of liquid monopropellant microthruster with spark ignition can be renewed, the decomposing of propellant also consumes more electricity than the liquid monopropellant microthrusters with the catalyst. Furthermore, a comparison for the advantages and disadvantages of the catalytic and spark ignition are listed in Table 4. In summary, liquid chemical propellants are traditionally attractive because they offer a relatively greater range of thrust level and the H_2_O_2_ used as liquid propellant is nontoxic. However, problems such as the stability and safety of chemical, materials compatibility, and shelf-life should also be paid attention when using chemical propellants.

## 3. Vaporizing Liquid Microthrusters (VLM)

Until now, VLMs studied by different groups could be classified into two categories: The internal heating and the external heating, according to the heating method. Generally, some microheaters are fabricated outside of the vaporization chamber and the internal liquid evaporation is accomplished though the heat conduction. Other microheaters are fabricated in the vaporization chamber and the resistive microheater needs to be connected with the outside electric power. The VLM is mostly fabricated using MEMS technologies on silicon or ceramic wafers based on electrothermal effect. It usually contains an inlet, a microchannel, a vaporizing chamber where the propellant is heated and vaporized by an internal or external microheater, and a micronozzle designed to accelerate the gasses to supersonic velocities. This process of phase transition increases the pressure in the vaporizing chamber and pushes the vapor exited through a micronozzle to produce thrust force. Although the VLM produces a relatively small range of thrust and specific impulse, this kind of microthruster has advantages of simple structure, low working voltagem and there is no pollution with water as propellants. Thus, many works researched on the VLM have been done in recent years.

### 3.1. VLM with Internal Microheater

**Figure 31:**
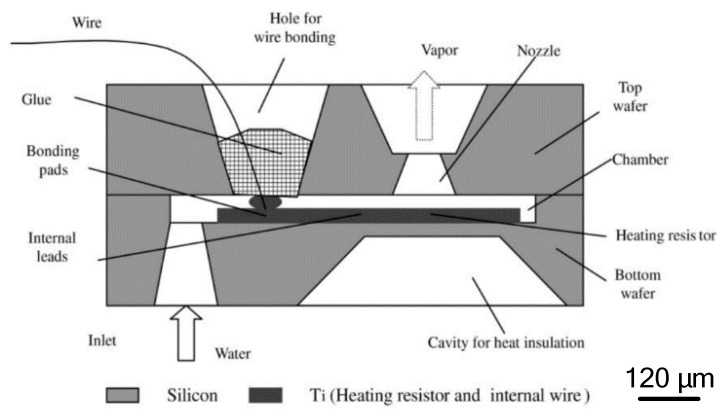
Schematic view of the vaporizing liquid microthruster (VLM) with an internal microheater designed by Ye et al. [75] Copyright © 2001, Elsevier Science B.V. With permission of Elsevier.

**Figure 32:**
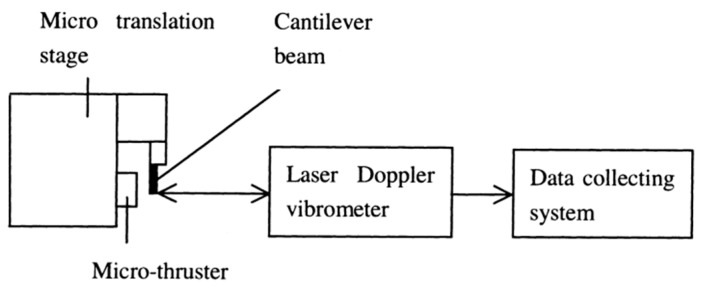
Schematic view of the VLM with measurement system designed by Ye et al. [75] Copyright © 2001, Elsevier Science B.V. With permission of Elsevier.

The VLM with internal microheater fabricated in the vaporizing chamber make the liquid contact with the microheater directly, which has the advantage of reducing the heat loss. However, it is somewhat complex for fabricating a VLM structure and connecting with an external wire. The first VLM based on MEMS was developed by Muller et al. in 1997 [74] followed by many studies on the VLM. Ye et al. [75] proposed a VLM earlier in 2001, which vaporized water into high-pressure gas by electric pulses. The microthruster fabricated on two silicon wafers contains a microresistor, a vaporizing chamber, a propellant inlet, a micronozzle, and a microchannel, as shown in Figure 31. The top and the bottom chip were bonded together by glue to form a microthruster chip. The metal Ti heater was fabricated in the vaporizing chamber and connected with the outside wires. The VLM thrust measurement system was designed as shown in Figure 32. When the microthruster was operating, the gas which dashes out through the nozzle hits the free end of the beam and causes it to deflect. Then, the approximate thrust of the microthruster can be calculated by measuring the displacement of the free end of the cantilever beam. The thrust of the microthruster was 2.9 μN with an input pulse power of 30 W.

**Figure 33:**
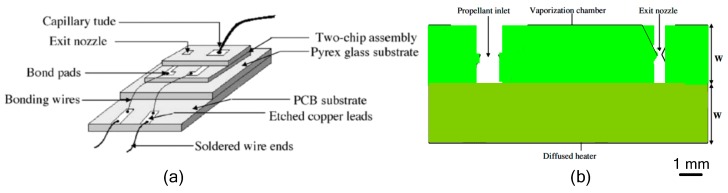
(**a**) Appearance structure diagram of VLM test device. [77] Copyright© 2005, Elsevier B.V. With permission of Elsevier; (**b**) cross-sectional view of the VLM. Figure modified from Ref. [76].

**Figure 34:**
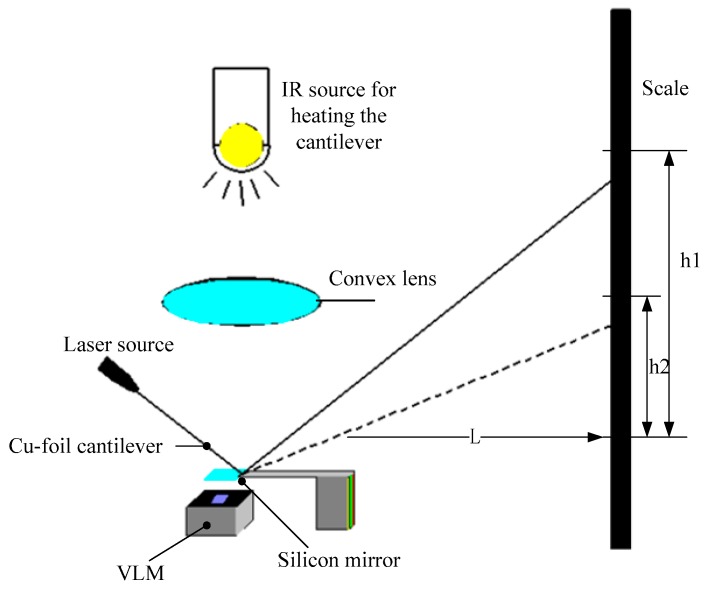
A schematic of the thrust measurement setup. Figure modified from Ref. [76].

Subsequently, Maurya et al. [76] developed a silicon-based VLM with an integrated p-diffused microheater and two bonded micromachined chips. The thrust produced ranged from 5 μN to 120 μN at a flow rate of 1.6 μL/s with a heater power ranged from 1.0 W to 2.4 W [77]. The internal heater was fabricated on the bottom silicon substrate by diffusing boron, and the p-diffused resistive heater was passivated by thermally grown silicon dioxide. A schematic of the appearance of the VLM test device and the cross-sectional view of the VLM are shown in Figure 33. The top wafer contains an inlet, an upper part of the vaporizing chamber, and an exit nozzle. The bottom wafer consists of a p-diffused meanderline resistor, a V-groove microchannel, and the other part of vaporizing chamber. Then, the adhesive bonding technique was employed. A schematic of the thrust measurement setup is shown in Figure 34. A small deflection of the cantilever of the VLM caused a small bending of the cantilever and the light spot on the screen moved. The lamp-and-scale arrangement effectively magnifies the deflection by several orders of magnitude. Then, the thrust was calculated by a series of relations

**Figure 35:**
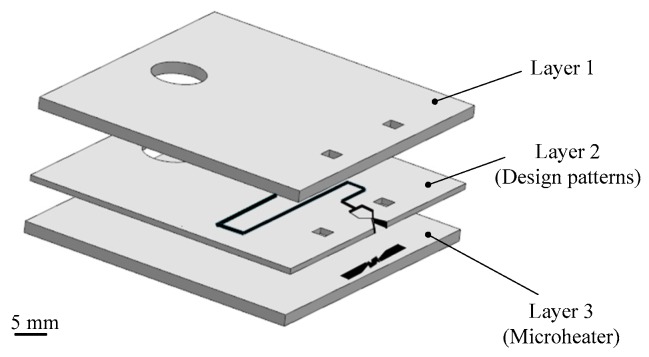
Exploded structure design of the high-temperature co-fired ceramic (HTCC) VLM. Figure modified from Ref. [80].

**Figure 36:**
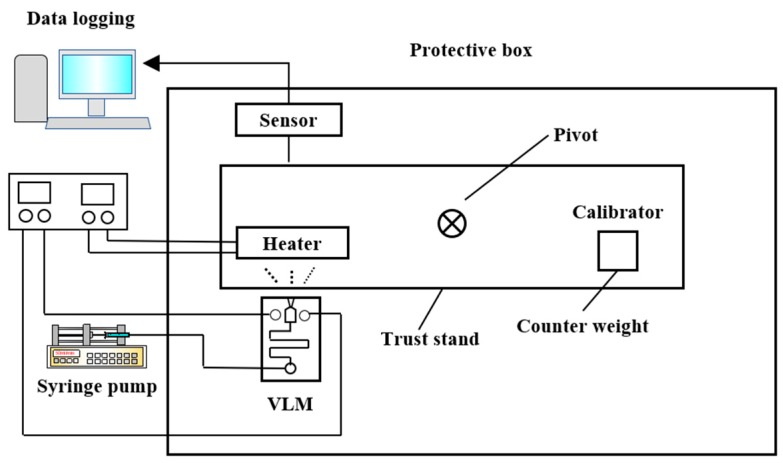
Schematic diagram of the torsional thrust stand measurement system. Figure modified from Ref. [80].

Besides silicon being used as the substrate material of VLM, ceramic has also been used in the fabrication of the VLM and microsystem [78]. LTCC technology has been successfully used for the first time in the designing of VLM in the study of Karthikeyan et al. [79]. After the experimental testing, the VLM with a nozzle throat size of 220 μm × 200 μm produced a thrust ranged from 34 μN to 68 μN and an average specific impulse ranged from 3.4 s to 6.9 s at a flow rate of 1 mg/s with an input power ranged from 7.1 W to 9.2 W. In addition, Cheah et al. [80] reported a study of a MEMS-scaled VLM fabricated based on the high-temperature co-fired ceramic (HTCC) technology. The VLM consisted of an injector, a vaporizing chamber, a micronozzle, and a microheater. The structure design of the proposed VLM is shown in Figure 35. The temperature was ramped at a higher rate of 6 °C/min to 1500 °C and a dwell time of 90 min for sintering. Finally, the furnace was cooled down to room temperature. The entire microheater was directly exposed to the vaporizing chamber, which maximizes efficiency of the heat transfer between the liquid propellant and the microheater. A block diagram of the thrust measurement system is shown in Figure 36. It consists of an aluminum alloy beam of 60 cm long supported by a flexural pivot (6012-400, Riverhawk, New Hartford, NY, USA), a high precision optical linear displacement sensor (ILD 2300-2, MicroEpsilon, Freistaat Bayern, Germany), an electrostatic calibrator, and a plexiglass protective box to reduce disturbances due to the external environment. Nevertheless, the influence of steam liquefied water droplets on the measurement accuracy is inevitable. A maximum thrust of 633.5 µN was reached at a flow rate of 1 µL/s.

### 3.2. VLM with External Microheater

**Figure 37:**
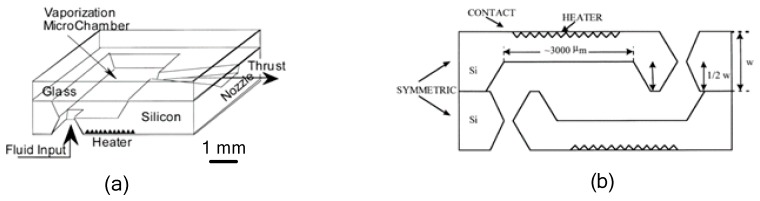
Diagram of two different microthrusters. (**a**) Side exit nozzle and (**b**) top exit nozzle. [81] Copyright © 2000, Elsevier Science S.A. With permission of Elsevier.

**Figure 38:**
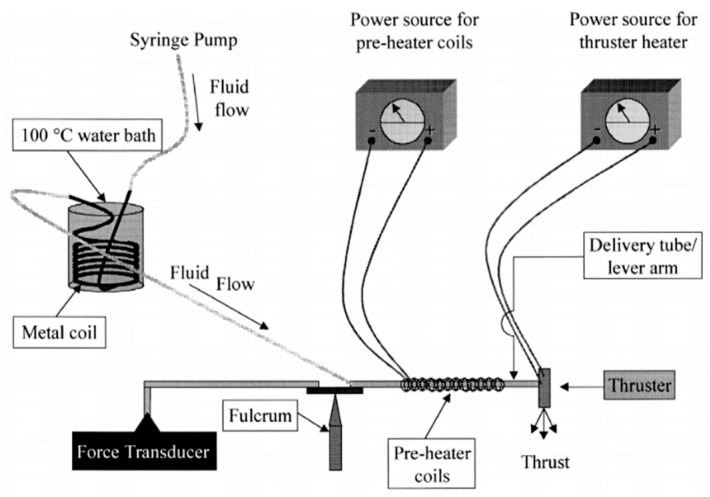
Schematic diagram of thrust measurement system. [81] Copyright © 2000, Elsevier Science S.A. With permission of Elsevier.

Although, the VLM with external microheater consumes more heat for heat conduction compared with the VLM with internal microheater, the microheater fabricated outside the chamber is more easily achieved. Mukerjee et al. [81] firstly designed a VLM with an external microheater, and two different micronozzle structure designs were presented: A side exit micronozzle structure design and a top exit micronozzle structure design, as shown in Figure 37. In the VLM with side exit micronozzle structure design, glass was used to seal the microchambers on the silicon substrate. The wafer was anodically bonded (Karl Suss Anodic Bonder SB-6) to a glass wafer Pyrex (#7740). In addition, the condition of fluid flow and vaporization in the microchamber can be visually observed from a window during the operation of VLM. In the top exit nozzle design, silicon-to-silicon fusion bonding was adopted. Two identical wafers were aligned and then brought into intimate contact and pressed together to eliminate voids. The pair was annealed for 10 min at 1000 °C in nitrogen followed by 50 min in dry oxygen. Then, two microheaters were placed on the surface of the upper and lower silicon wafers, respectively. The schematic diagram of thrust measurement system is shown in Figure 38. The VLM was fixed at one end of a rigid arm, in the center of which rested on a knife-edge fulcrum. The liquid flowed from the syringe pump and was preheated with a water bath and a heating coil. The resultant thrust was measured at the opposite end of the arm by using a calibrated microbalance with 10 mg resolution (American Scientific Products SP-182). Thrust ranging from 0.15 mN to a maximum value of 0.46 mN was produced at an input power of 10.8W during experimental testing.

**Figure 39:**
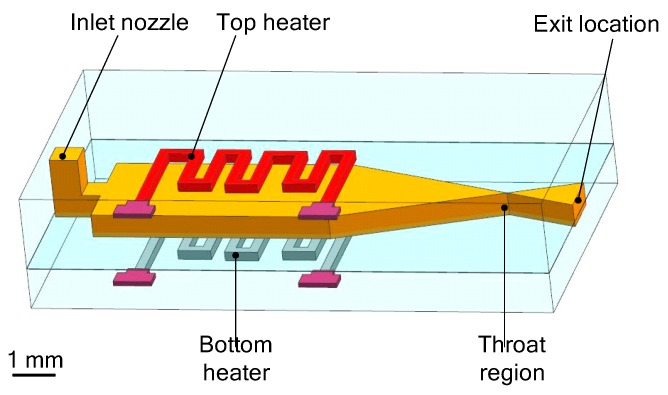
3D structure design of a VLM with top and bottom microheaters. Figure modified from Ref. [82].

**Figure 40:**
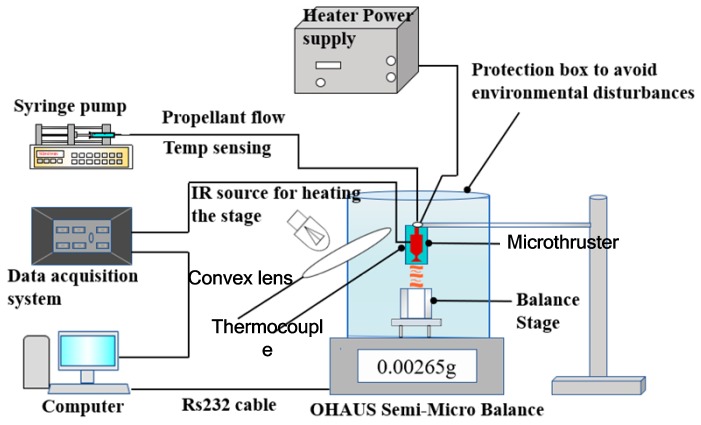
Schematic of the thrust measurement setup using a semi-microbalance. Figure modified from Ref. [82].

The microheaters located both on the top and bottom surface of the device were also presented in the study of Kundu et al. [82]. The VLM consists of an inlet channel, a microchamber, and a converging–diverging (C–D) planar exit nozzle integrated in two micromachined, bonded silicon chips by adhesive glue. A 3D structure design of the VLM is shown in Figure 39. The schematic of the thrust measurement system using a semi-microbalance to evaluate the VLM performance is shown in Figure 40. A high-sensitivity laboratory balance (DV215CD, OHAUS Discovery Semi-micro Dual Range Balance) was adopted for the measurement. In addition, the condensation of the liquid vapor on the balance stage was avoided by infrared heating using an IR bulb. However, if the microthruster works for a long time, some of the vapor liquefaction will still have an influence on the results. The thrust of the VLM could reach 1 mN at a flow rate of 2.04 mg/s and a maximum heater power of 3.6 W with a microthroat area of 130 μm × 100 μm.

**Figure 41:**
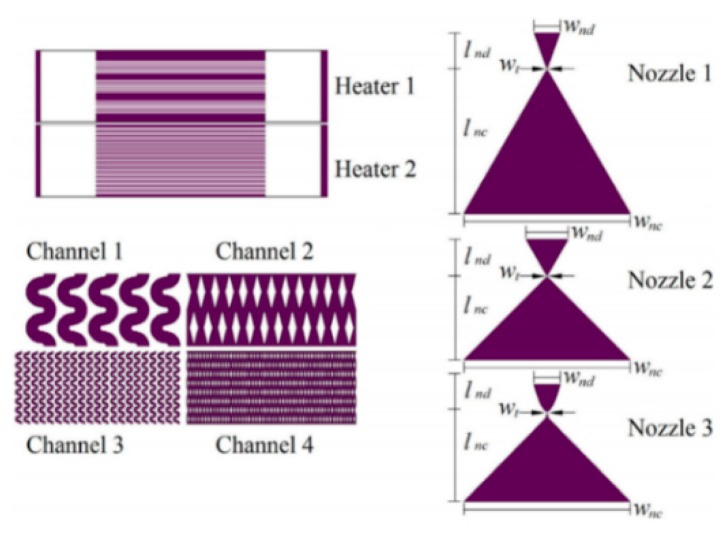
Schematic view of different structure designs for each part of VLM [83]. Copyright © 2017, Elsevier B.V. With permission of Elsevier.

**Figure 42:**
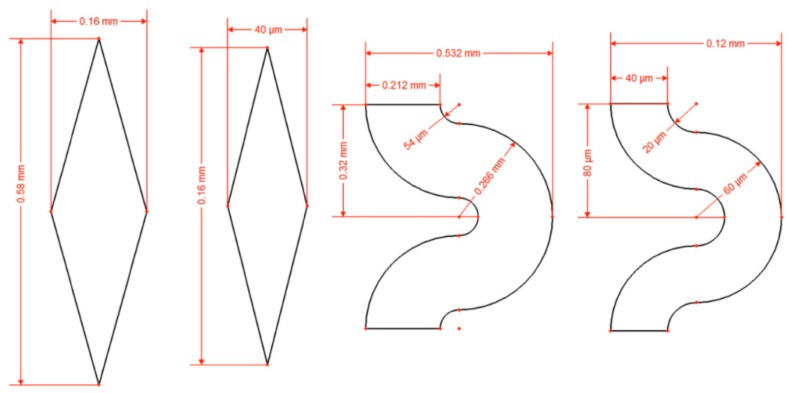
Different design parameters of the channels. From left to right: Large diamonds, small diamonds, large serpentine, and small serpentine [83]. Copyright © 2017, Elsevier B.V. With permission of Elsevier.

Each part of the microthruster, such as the micronozzle, the vaporizing chamber, and the microheater, has an impact on the performance of VLM. Silva et al. [83] mainly proposed different design for each part of VLM and analyzed the performance of microthruster by simulation. There are three types of nozzle, four types of channels, and two types of heaters designed, as shown in Figure 30. The design parameters of the channels are shown in Figure 41. The design parameters of the channels are shown in Figure 42. In addition, three types of nozzle have different area ratios (i.e., the ratio between the exit area of the nozzle and the throat area); the long nozzle has an area ratio of approximately 11, the wide nozzle 17, and the bell nozzle 11. The thrust generated with the same area ratio of the long nozzle and the bell nozzle ranged from 0.75 mN to 3.79 mN and specific impulse ranged from 105 s to 113 s. The VLM with wide nozzle generated a relative higher thrust that ranged from 0.77 mN to 3.86 mN and specific impulse ranged from 107 s to 115 s.

**Figure 43:**
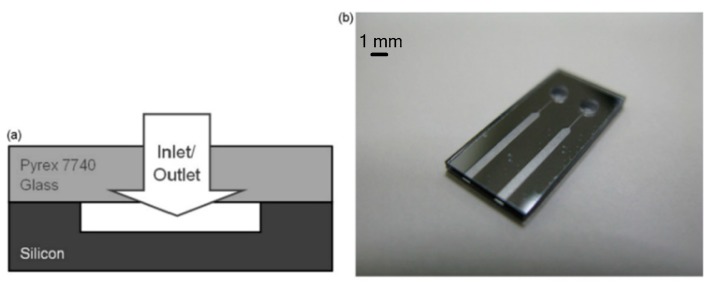
The VLM chip consists of a Pyrex glass and a silicon: (**a**) The cross-section of one channel, (**b**) the picture of the VLM with an outlet width of 1000 µm. [84] Copyright © 2009, Elsevier B.V. With permission of Elsevier.

Chen et al. [84] also designed a VLM and simulated the performance of the thruster. In order to investigate the flow characteristics of VLM, a 3D model was constructed and simulated. The prototype of the VLM consists of a Pyrex glass wafer and a silicon wafer with the VLM structure of two inlet and two parallel channels, as shown in Figure 43. The Pyrex7740 glass with an injecting hole was served as the top cover to be bound with Si wafer using anodic bonding technique under an applied voltage of 700 V at a process temperature of 450 °C for 15 min. The channels were designed as outlets with the cross-section of 1 mm × 100 µm and the thrust obtained ranged from 1 mN to 6 mN approximately, which was calculated by simulation.

### 3.3. Summary of VLM

In conclusion, all these VLMs adopted electrical resistor as heat sources. The fabrication of most VLMs was carried on a silicon or ceramic substrate, which has advantages of simple structure and simplified fabrication process. Additionally, the problem of easy decomposition of liquid chemical propellant is also avoided by using water as propellant. However, the performance of the VLM is lower than the microthruster with liquid chemical propellants, which is reflected in thrust force and specific impulse. Table 5 presents the data for comparison extracted from the references of VLMs. In addition, a comparison for the advantages and disadvantages of external and internal microheater are listed in Table 6.

## 4. Plasma Microthruster

The plasma microthruster adopted from MEMS technology mainly contains the liquid/solid propellant pulsed plasma microthruster [85] and electrothermal plasma microthruster [86,87]. This section mainly introduces the characters of different types of plasma microthrusters presented by different groups.

### 4.1. Electrothermal Plasma Microthruster

The electrothermal plasma microthruster works by the principle of low-power plasma discharging, which can be generated by using radio frequency (RF) power or microwave power, and the propellant is heated by the heat generated by such a discharge process. Therefore, electrothermal plasma microthruster mainly includes microwave electrothermal thrusters (METs) and RF electrothermal thrusters (RFETs). Additionally, METs and RFETs have similar performance in that it needs less than 10 W powers to generate thrust in the order of mNs and specific impulses up to 85 s for using Ar. The electrothermal plasma microthruster usually adopted gas propellants, such as Ar, H_2_, and He. The gas is heated by the charge exchange collisions and ambipolar flow of plasma to create a form of electrothermal thruster [88]. Electrothermal plasma microthruster also has advantages of small volume, low cost, and lightweight.

**Figure 44:**
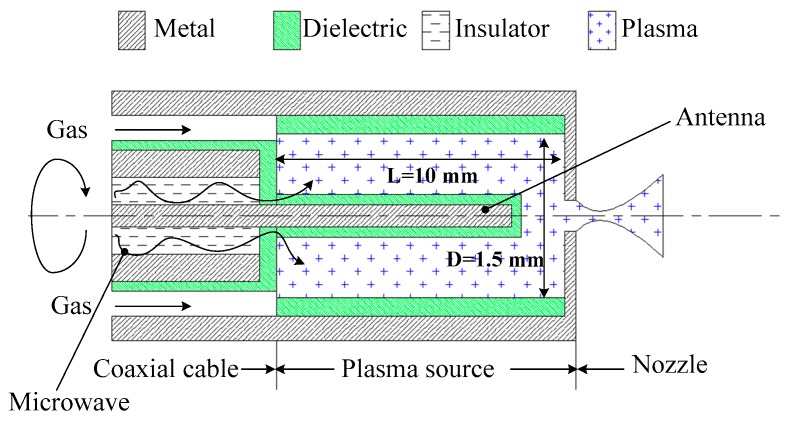
Schematic diagram of the microwave electrothermal thrusters (MET) device. Figure modified from Ref. [89].

Takahashi et al. [89] investigated a MET using azimuthally symmetric. The microthruster consists of a rod antenna on axis, a micro plasma source, and a converging–diverging (Laval) micronozzle. The schematic of the MET is shown in Figure 44. The micronozzle was fabricated in a quartz plate 1 mm thick by using a micromachining process with a diamond drill. The plasma source consisted of a dielectric chamber and a metal rod antenna covered with a dielectric envelope, which produces high-temperature plasmas at around atmospheric pressures. The Laval nozzle converts the high thermal energy of plasmas into directional kinetic energy of supersonic plasma flows to generate a thrust. Ar was adopted as propellant gas with flow rates of 10–70 sccm under the condition of 4.0 GHz microwaves excited and input powers less than 6 W. The thrusts generated ranged from 0.2 mN to 1.4 mN and the specific impulses obtained ranged from 50 s to 80 s. Subsequently, a study of H_2_ and He adopted as propellant for plasma microthrusters was presented [90]. Similarly, the thrusts ranged from 0.04 mN to 0.51 mN and the specific impulses ranged from 150 s to 270 s were obtained when the source pressure ranged from 0.5 kPa to12 kPa at flow rates of 2–70 sccm.

**Figure 45:**
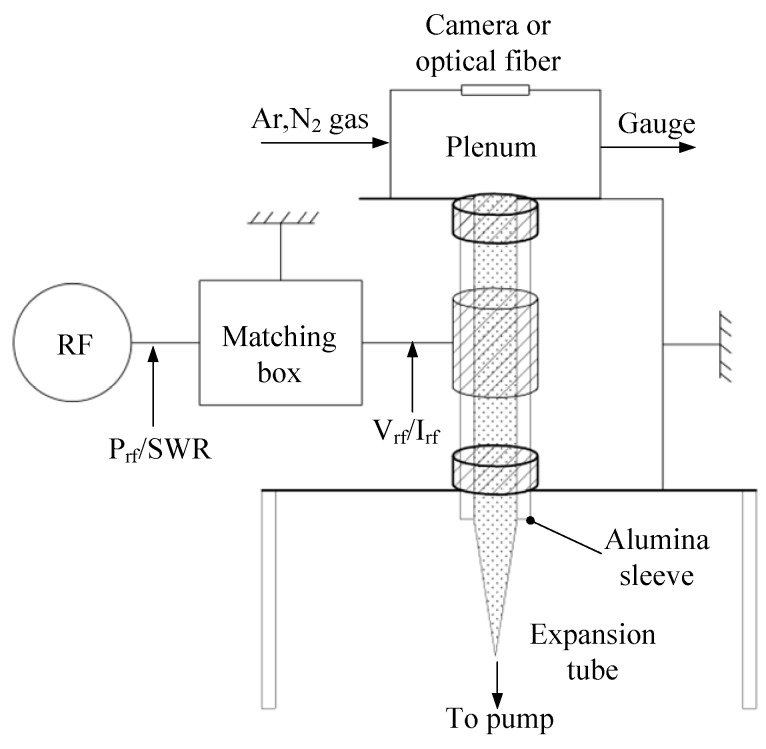
Schematic diagram of radio frequency electrothermal thrusters (RFET) experimental system setup. Figure modified from Ref. [91].

Aiming at the application in the Pocket Rocket, Greig et al. [91,92] developed a microthruster of RFET based around gas heating from ion-neutral charge exchange collisions. The schematic diagram of the device is shown in Figure 45. The device consists of an expansion tube and an alumina sleeve.

### 4.2. Liquid/Solid Propellant Pulsed Plasma Microthruster

The pulse plasma microthruster accelerates a small amount of plasma to a high discharge velocity by the rapid discharge of the energy from an energy storage capacitor. Variable thrust of a microthruster can be realized by varying the pulse rate. Pulsed plasma microthruster is one of the promising micropropulsion devices based on electric driven and it has the structure characteristics of simple, light, and small volume. Moreover, pulsed plasma microthruster can generate an accurate thrust and a relatively large specific impulse of ∼4300 s with optional pulse interval.

**Figure 46:**
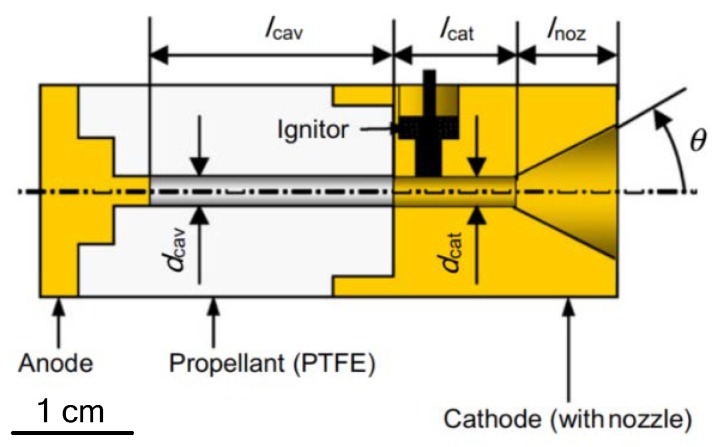
Schematic of the coaxial pulsed plasma microthruster. [94] Copyright © 2008, Elsevier Ltd. With permission of Elsevier.

Guman et al. [93], relatively early, presented the design of a solid propellant pulsed plasma microthruster to meet specific mission requirements. The solid propellant pulsed plasma microthruster utilized poly-tetrafluoro ethylene (PTFE) as propellant, which has some advantages, such as simple structure, high reliability, and low electrical power for its operation. Aoyagi et al. [94] designed a pulsed plasma microthruster with s coaxial electrode that consisted of an anode, a cathode, propellant, and an igniter, as shown in Figure 46. The results of experiments showed that the divergent nozzle and cathode diameter influenced thrust performances and relatively larger impulse bit ranged from 300 μN·s to 800 μN·s was realized, with a total impulse of 40 N·s achieved.

**Figure 47:**
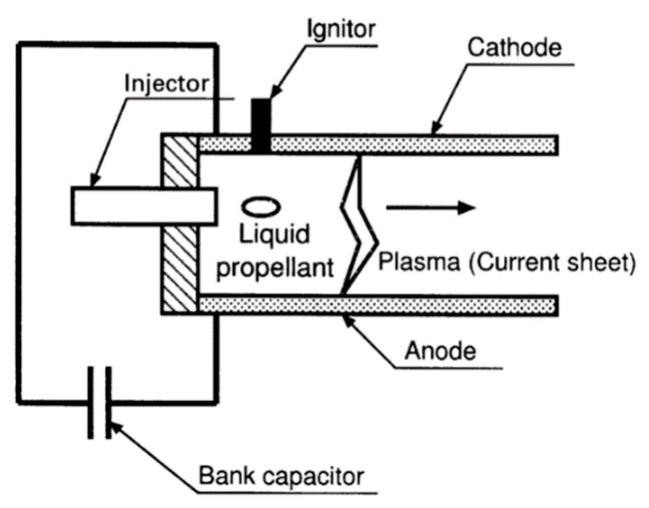
Schematic of liquid propellant-pulsed plasma thruster (LP-PPT) designed by Kakami. [96] Copyright © 2004, Elsevier Ltd. With permission of Elsevier.

Generally, most pulsed plasma microthrusters are operated on electromagnetic forces to accelerate solid propellant to generate a thrust. It also has been proposed that a discharge can be initiated in a pulsed plasma microthruster at an under-voltage by shining an IR laser pulse on the backplate of microthruster [95]. Although pulsed plasma microthrusters are a promising microthruster because of characteristics such as being compact and light, there are also some disadvantages such as contamination, low thrust performance, and non-uniform consumption of propellant [96]. Thus, water or alcohol has been used as a propellant in the developing of liquid propellant-pulsed plasma thruster (LP-PPT) to solve these problems. Kakami et al. [96] designed a LP-PPT with parallel electrodes as an igniter in order to allow the LP-PPT to be operated with a wider electrode gap. The LP-PPT achieved a thrust efficiency of 13% and reached a specific impulse of 4300 s at energy of 20 J. The schematic of the LP-PPT is shown in Figure 47, and includes an intermittent injector, an igniter, and electrodes. The liquid propellant droplets are converted into plasma by the discharge current, subsequently, which will be accelerated electromagnetically and electrothermally. In conclusion, thrust is generated by the plasma propagation. The advantage of the LP-PPT is that there is no requirement for a high-pressure reservoir and cryogenic devices. Additionally, requirements of low leakage, low power consumption, compactness, and lightness must be satisfied for the injectors of the LP-PPT.

### 4.3. Summary and Comparison

The advantages and disadvantages of the electrothermal plasma microthruster and the liquid/solid propellant pulsed plasma microthruster are summarized in Table 7. For electrothermal plasma microthruster, gas was usually adopted as propellant. Therefore, it is necessary for storage of the propellants to have a high-pressure reservoir or cryogenic devices. For liquid/solid propellant-pulsed plasma microthrusters, there is not a problem in the storage of the propellants generally. However, it also requires low leakage, low power consumption, compactness, and lightness for the injectors. In conclusion, for both two types of microthruster, they have the advantages of small volume, simple structure, and light weight.

## 5. Colloid Microthruster

Colloid microthrusters work by the electrostatic atomization, charging, and acceleration of the propellant [97]. The colloid microthrusters can generate thrusts ranged from 1 µN to 20 µN and specific impulses ranged from 500 s to 1300 s. The most attractive characteristics of colloid microthrusters is that the thrust can be generated from a fraction of a few µN range with the same engine and propellant, which also allows one engine to perform a span of missions from precise disturbance cancellation to prime propulsion [98].

**Figure 48:**
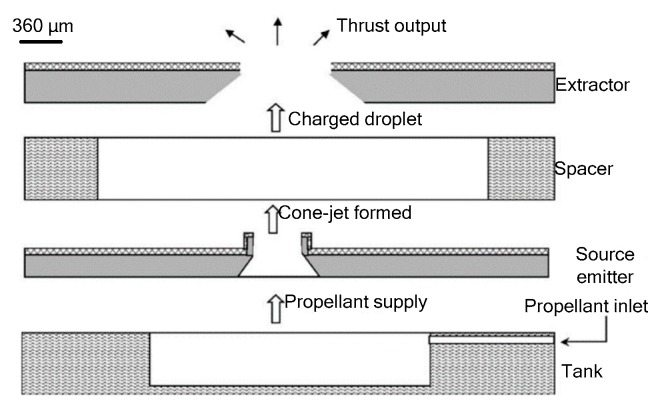
Schematic diagram of colloid microthruster designed by Xiong et al. [99] Copyright © 2004, Elsevier B.V. With permission of Elsevier.

Xiong et al. [99] developed a sandwich structure colloid microthruster with bulk silicon processing. As shown in Figure 48, the microthruster consists of a propellant tank, an extractor, a source emitter, and a space between extractor and source emitter. Liquid propellant was driven by capillary force through the microchannel to the tip of the source emitter. Then, liquid was broken up into charged droplets by the intense electric field and accelerated to exit the extractor to produce a thrust. The source emitter and extractor were fabricated in two wafers separately by different processing and were bonded together to form a microthruster device. The experiments verified that thrust ranging from 1.20 µN to 4.85 µN was obtained when the applied voltage increased from 1400 V to 2800 V. The advantages of the microthruster are that the power unit of micropropulsion was simple and the thrust generated is as small as µN. Xiong et al. also fabricated an improved colloid microthruster with a silicon micropump integrated and PCB based [100]. They adopted a precision current vortex displacement sensor to transform the displacement into an electrical signal with a sensitivity of 8 mV/µm. A maximum thrust produced by the improved microthruster reached approximately 2.56 µN under the driving voltage of 3500 V.

A research group from the Space Propulsion Lab at MIT developed a colloid microthruster array [101]. Furthermore, an addressable colloid microthruster array was designed by Paine and Gabriel with an expected minimum thrust of 0.1 µN [102], in which the source emitter and extractor were designed in the same wafer. Colloid thrusters also were developed for the ST7-DRS (Space Technology 7 Disturbance Reduction System) and LISA (Laser Interferometer Space Antenna) Missions [103,104,105]. However, further developments were limited to the packaging problems caused by high starting and working voltages ranging from 5 kV to 10 kV [106]. Accordingly, the characters of colloid microthruster are large driving voltages but low thrust.

## 6. Electrospray Microthruster

**Figure 49:**
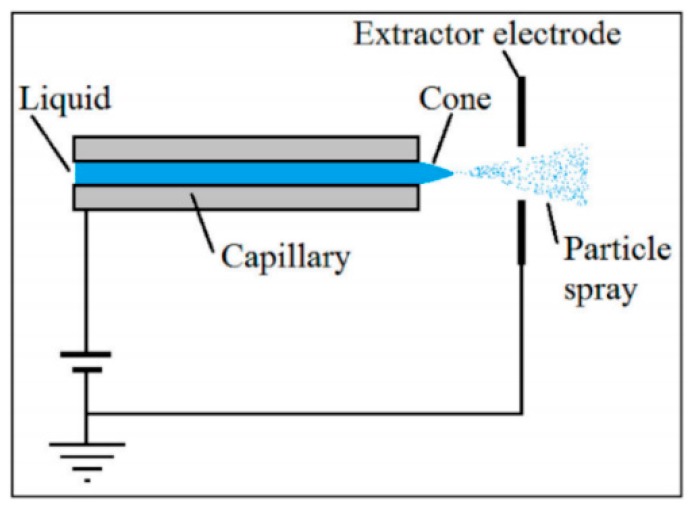
Schematic diagram of an electrospray microthruster [110]. Copyright © 2017, IAA. With permission of Elsevier.

Electrospray microthruster is an atomizer that produces thrust by emitting a spray of particles created by what is called a Taylor cone [107], in which charged liquid droplets or ions are extracted from an emitter via an applied electric field. Thus, electrospray thrusters can produce a high specific impulse at low flow rate, but a low thrust. A schematic diagram of an electrospray microthruster is shown in Figure 49. The propellant can be either an ionic liquid or mixture or a liquid metal. The emitters could be incremented with an accelerator grid after the extractor to further increase the exit velocity of the particles [108]. The electrospray microthrusters have been acknowledged as a promising technology to provide micropropulsion capabilities to small spacecraft, due to their relative high performance and simplicity [109].

**Figure 50:**
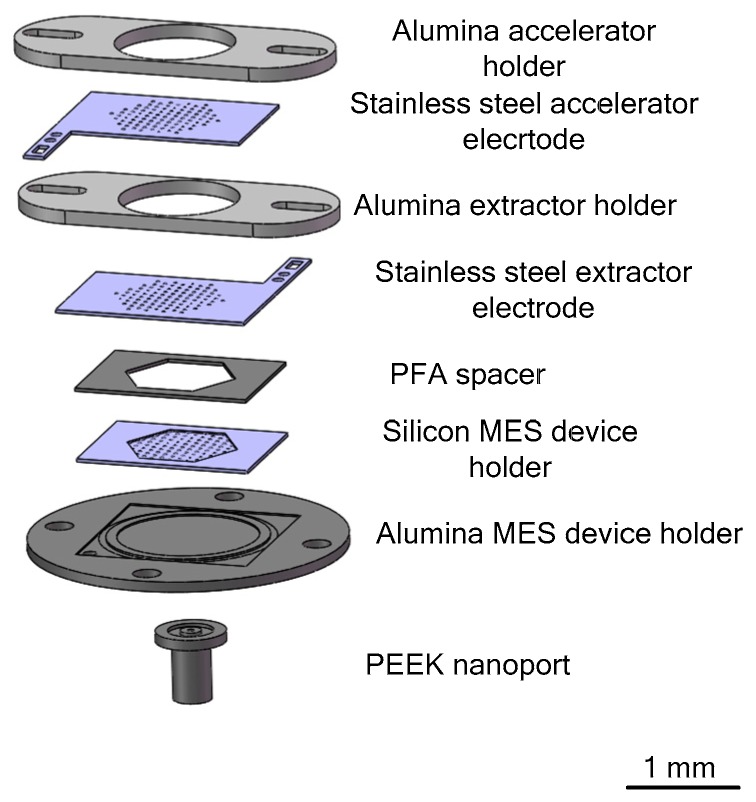
Exploded view of a MES microthruster assembly designed by Lenguito et al. Figure modified from Ref. [111].

Lenguito et al. [111] reported a multiplexed electrospray (MES) microthruster, which covers a wide range of specific impulse and generated thrust at high (> 50%) propulsion efficiency by emitting fast nanodroplets. The MES microthruster consists of a nozzle unit and two electrodes, which include an extractor electrode and an accelerator electrode with optimal interelectrode insulation in order to make the applied voltage maximize. Figure 50 shows an exploded view of the MES microthruster assembly. The performance of different devices with 7, 37, and 91 nozzles were examined, respectively. The 37-MES microthruster produced a thrust of 31.10 μN at a voltage of 7.56 kV and specific impulse reached 1870 s. Moreover, the 91-MES devices produced a thrust of 65.20 μN and specific impulse reached 1140 s at a voltage of 7.35 kV.

Moreover, in the study of Berg et al. [112], experimental measurements of the electrospray emission and beam properties of the double salt ionic liquid propellant [Emim][EtSO_4_]-HAN were described. A voltage of 3400 V was necessary for exhibiting stable electrospray emission. The highest specific impulse of 412.37 s was achieved in experiment and the thrust was 8.71 μN.

## 7. Free Molecular Micro-Resistojet (FMMR)

**Figure 51:**
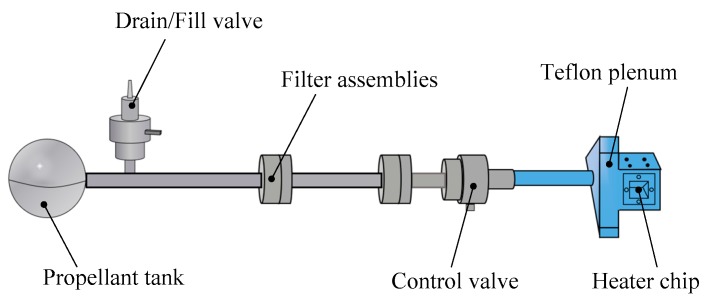
Optimized nanosatellite FMMR propulsion system. Figure modified from Ref. [116].

The FMMR is an electrothermal micropropulsion system [113] and an electrically heated surface is the main heating method to provide heat when the propellants flow through the FMMR. The FMMR mainly consists of three parts: The heater chip fabricated by MEMS, the flow control, and the propellant storage tank [114,115,116], as shown in Figure 51. The thruster system is operated on the vapor pressure of water and the water is stored in the states of either liquid or solid (depending on the temperature of internal satellite) [117]. The propellant gas arrives in the Teflon plenum after passing from the propellant storage tank through the propellant feed system. The thrust is generated by expelling the propellant gas in the plenum through a series of the expansion slots in the heater chip fabricated by MEMS technology. The main factor affecting the performance of FMMR is heat loss. The heating efficiency of the resistojets relied on the contacting of the propellant and the heated walls or a coil, but this will lead to short ended lifetimes of FMMR device because of thermal fatigue. However, there is no heat conduction and heat convection between the FMMR and the space environment when FMMR is used in space spacecraft, and the power required by FMMR will be greatly reduced. Thus, the FMMR is designed as a micropropousion system for attitude control of nanospacecarft. The FMMR can also provide a thrust level ranged from several μN to a few mN. The FMMR thruster based on MEMS has the advantages of simple structure, lightweight, fast response, low cost, high reliability, high integration capability [118], and so on.

## 8. Cold Gas Microthrusters (CGM)

**Figure 52:**
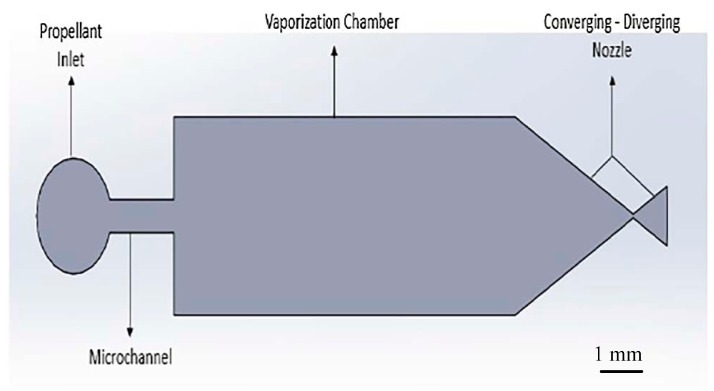
Example of a cold gas microthruster [122]. Copyright © 2018, Elsevier Ltd. With permission of Elsevier.

**Figure 53:**
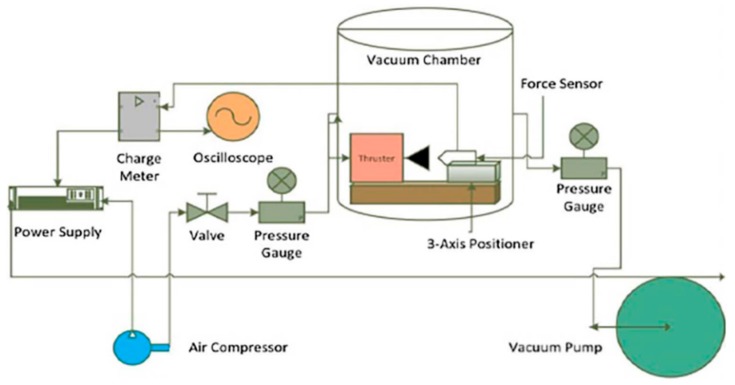
Schematic diagram of the experimental setup of a cold gas microthruster [122]. Copyright © 2018, Elsevier Ltd. With permission of Elsevier.

The cold gas microthruster is also very suitable for small spacecraft and micro/nano satellites [119]. This kind of microthrusters uses a pressurized inert gas as the propellant stored in the states of liquid, gaseous, or solid phase. The work principle is that gas will accelerate to high velocities producing thrust when passes through a micronozzle. For example, one of the CGM is designed as shown in Figure 52. It has the advantages of simple structure, reliability, and ease of miniaturization. However, cold gas microthusters also need cryogenic and high-pressure storage systems for the storage of gas propellants. A Kistler force sensor (model 9207) and a charge meter (model 5015) were used to measure thrust produced by the microthruster. A schematic diagram of the experimental setup is shown in Figure 53.

Kohler et al. [120] presented a hybrid CGM system, which included three different micromachined parts: A nozzle unit, four independent piezoelectric proportional valves, and two particle filters. It was designed to deliver thrust ranged from 0.1 mN to 10.0 mN and the specific impulse of 45 s was reached for nitrogen propellant. The single crystalline silicon was adopted as primary material to fabricate the different parts of the hybrid CGM system, which was shaped by DRIE. Rhee et al. [121] developed a CGM system and the CGM has proven to be reliable when tested on spacecraft. Moreover, it also has the potential to be miniaturized to meet the requirements of CubeSats. Ranjan et al. [122] reported on CGM development and investigated the gas feeding in a liquid fueled microthruster. The thrust ranged from 0.80 mN to 2.24 mN were obtained under vacuum conditions.

The CGM has the simple form of micropropulsion where an inert gas can be expelled through a nozzle. The CGM system also has advantages of non-toxicity, easy to use, and no chemical decomposition concerns. However, thrust of CGM produced per kilogram of propellant is relatively low due to no combustion or heating of the gas.

## 9. Conclusions

In this paper, the development of the MEMS-based microthrusters is summarized. The structure and characteristics of several typical microthrusters are described and then the performance of these micropropulsion systems are compared and analyzed. According to the working principles, various microthrusters have different performance indexes. Among the advantages of MEMS technology are the miniaturization and integration. Moreover, adopting MEMS technology can also reduce the costs by the batch processing of items and has the ability to operate in micrometer range [123].

Table 8 presents a total comparison of all types of microthruster. In general, the SPM has advantages of simple structure, high propellant stability, and high reliability. Additionally, the SPM also has characteristics such as no leakage of propellant and no frictional forces because of inherent to moving parts. However, the main disadvantages of the SPM are one-shot use. The liquid monopropellant microthruster has a relatively greater range of specific impulse and thrust level, which has a small demand for electricity. Nevertheless, it also has the disadvantage that the propellants are easy to decompose. The VLM have advantages of simple structure, low voltage, and easy to fabricate. However, they also have disadvantages of relatively low specific impulse. The plasma microthruster has advantages of low-volume, low-cost, relative larger specific impulse, and low-weight, but it needs higher operating voltage. The electrospray microthruster can produce a larger specific impulse but thrust is relatively low. Although the colloid microthruster can provide a relatively large specific impulse range, it also needs a larger working voltage. In the operation of FMMR, the heat is mainly provided by contacting with heated walls or a coil, which is easy to be realized. The advantages of FMMR are low thrust noise, high thrust accuracy, and repeatability. However, this method of heating will lead to short lifetimes of the FMMR device due to thermal fatigue, which also require large power. In addition, the whole micropropulsion system is relatively bigger. Therefore, the scope of its application may be limited in some areas where space is particularly small. The CGM has the advantages of simple structure, reliability, low energy consumption, and easy to be miniaturized, but it has relatively low specific impulse and needs a high-pressure gas storage tank. There are also some problems such as large volume and weight, difficulty in preventing leakage, and so on

There are four kinds of bonding methods mainly used including adhesive bonding, anodic bonding, eutectic bonding, and LTCC/HTCC process bonding. Table 9 presents the comparison of these bonding methods.

To sum up, microthrusters can basically meet the needs of present micro-satellites in thrust requirement. Although microthrusters have been tested on some spacecraft according to the application prospects and strong needs, they are still in the stage of researching and developing as a whole and the technology is also not mature enough, requiring further study and testing. In the future, the MEMS microthruster will be the mainstream trend due to the advantages of high performance, small size, and low power consumption. Additionally, the MEMS microthruster will continue to be developed rapidly by strengthening the research on optimal design, MEMS manufacturing technology, fuel selection, testing, and so on. In our opinion, if the measurement devices of thrust force can be improved to ensure the measurement accuracy, it will be very helpful to the further development of the microthruster.

## Figures and Tables

**Table 1:** Classification of microthrusters.

Main Features	Categories	Types	Sections
Chemical fuel propellant	Solid propellant microthrusters (SPM) Liquid monopropellant microthruster	Vertical structure Planar structure	2.1.1 2.1.2
		With spark ignition With catalytic ignition	2.2.1 2.2.2
Electric driving	Vaporizing liquid microthruster (VLM)	Internal microheater	3.1
		External microheater	3.2
	Plasma microthruster	Electrothermal plasma microthruster	4.1
		Liquid/Solid propellant pulse plasma microthruster	4.2
	Colloid microthruster		5
	Electrospray microthruster		6
	Free molecule micro–resistojet (FMMR)		7
Gas propellant	Cold gas microthruster (CGM)		8

**Table 2:** The performance of SPM and SPM arrays studies by different researchers.

Researchers	Structure	Ignition	Propellant	Thrust(mN)	Specific Impulse (s)
Rossi et al. [34]	Vertical	Top	GAP/ZPP	0.3–22.0	N/A
Rossi et al. [53]	Vertical	Top	GAP/AP/Zr,ZPP	0.3–2.3	0.059–24.333
Briand et al. [32]	Vertical	Top	ZPP	N/A	N/A
Lee et al. [36]	Vertical	Top	LTNR	3840 (peak value)	62.3 (average)
Ru et al. [41]	Vertical	Top	Nano-Al/Cuo + 10 wt% NC	479–645	10.2–27.2
Liu et al. [46]	Vertical	Bottom	HTPB/AP	N/A	6.443–23.366
Zhang et al. [48]	Planar	Top	Gunpowder	130 (peak value)	2.68–14.65 (sea level)
Zhang et al. [49]	Planar	Top	Gunpowder	380 (peak value)	5.55–14.41 (sea level)
Chaalane et al. [50]	Planar	Top	DB + x % BP	0.1–3.5	N/A
Lewis et al. [26]	Vertical	Bottom	LTNR	N/A	0.1 (impulse/mN·s)
You et al. [52]	Planar	Bottom	HTPB/AP	N/A	0.004–0.011 (impulse/mN·s)
Sathiyanathan et al. [54]	Vertical	Top	GAP/AP	0.18–0.29	0.138–5.556
Shen et al. [55]	Planar	Bottom	LTNR	25 (peak value)	0.00025

Table. *Cont.* N/A indicates no available data.

**Table 3:** The comparison of various liquid monopropellant microthrusters.

Authors	Material	Electric Energy	Propellant	Catalyst/ Microheater	Input Flow Rate (mL/min)	Thrust (mN)	Specific Impulse (s)
Wu et al. [58]	Ceramic	45 V	HAN	Ag	N/A	100–200	32.3–64.5
Kuan et al. [66]	N/A	10–15 W	92 wt% H_2_O_2_	Ag	9.56	182	101
Cen and Xu [67]	Si-Pyrex glass	0–10 V	H_2_O_2_	Pt	0.09–0.44	2.0–6.5	65–105
Kundu et al. [69]	Si-Pyrex glass	44 J	50 wt% H_2_O_2_	MnO_2_	0.20–1.25 (mg/s)	0.3–1.1	80–180
Khaji et al. [71]	Ceramic	3.7 W	30 wt% H_2_O_2_	Pt/Al_2_O_3_	0.05	0.84–0.96	92–106
Huh and Kwon [72]	Glass	N/A	90 wt% H_2_O_2_ + Ethanol	Pt/Al_2_O_3_	1.7	30.2	77.6
Huh et al. [73]	Glass	N/A	90 wt% H_2_O_2_	Pt/Al_2_O_3_	3	48	70.4

Table. *Cont.* N/A indicates no available data.

**Table 4:** The comparison for the advantages and disadvantages of the catalytic and spark ignition.

Ignition Methods	Advantages	Disadvantages
Spark ignition	(1) Larger energy (2) The propellants can be renewed (3) Longer service life	(1) Connecting wires are complex (2) It consumes more electricity
Catalytic ignition	(1) A small demand for electricity (2) More convenient in fabrication	(1) The efficiency of propellant decomposition may be insufficient (2) Trouble in different propellants may require different catalysts (3) Shorter service life

**Table 5:** The comparison of various VLMs that have been reported.

Authors	Material	Microthroat Size (µm)	Input Flow Rate (mg/s)	Input Power (W)	Thrust (mN)	Specific Impulse (s)
Ye et al. [75]	Si	N/A	0.038	30	0.003	7.78
Maurya et al. [77]	Si	30 × 30	1.6	1.0–2.4	0.005–0.120	N/A
Karthikeyan et al. [79]	LTCC	220 × 220	1	7.1–9.2	0.034–0.068	3.42–6.90
Cheah et al. [80]	HTCC	250 × 125	0.2–1.6	4.01	0.634	31
Mukerjee et al. [81]	Si	N/A	8.83	10.8	0.15–0.46	5.33
Kundu et al. [82]	Si		0.20–2.04	1.4–3.6	0.15–1.01	50–105
Silva et al. [83]	Si		0.55–0.83 (simulation)	7.29–8.76 (simulation)	0.67–0.98 (simulation)	119.8–124.0 (simulation)
Chen et al. [84]	Si	1000 × 100	2.08–16.67 (simulation)	N/A	1–6 (simulation)	N/A

Table. *Cont.* N/A indicates no available data.

**Table 6:** The comparison of external and internal microheaters.

Heating Methods	Advantages	Disadvantages
External microheater	Fabrication of structure is easier	Consumes more heat for heat conduction
Internal microheater	Contacting with the water directly can reduce the heat loss	Complex for fabricating and connecting with external wire

**Table 7:** The comparison of the electrothermal plasma microthruster and the liquid/solid propellant-pulsed plasma microthruster.

Types of Microthruster	Advantages	Disadvantages
Electrothermal	Produces high temperature plasmas at around atmospheric pressures by using RF power or microwave power	(1) Lower specific impulses (2) Needs a high-pressure reservoir or cryogenic devices
Liquid/Solid propellant pulsed plasma	(1) Produced a relatively higher specific impulse (2) No requires for a high-pressure reservoir and cryogenic devices	(1) Low working frequency and propulsion efficiency (2) Contamination and non-uniform consumption of propellant

**Table 8:** A total comparison of all types of microthruster.

Types of Microthruster	Performances	Advantages	Disadvantages
(1) Solid propellant microthrusters (SPM)	High thrust (>100 mN) Low specific impulse (< 100 s)	Easy propellant loading, no leakage of propellant, low cost and power consumption	One-shot use, lack of restart ability, and combustion instability
(2) Liquid monopropellant microthruster	High thrust (0.3–200 mN) High specific impulse (5–180 s)	Small demand for electricity, simplified fabrication and low cost, non-toxic combustion products.	Propellants are easily to decompose, heat preservation, and ventilation of storage tanks.
(3) Vaporizing liquid microthruster (VLM)	Low thrust (0.03–1 mN) Low specific impulse (3.42 –105 s)	Simple structure, low voltage, low cost and easy to fabricate, no pollution of propellants	Too difficult to reach more than 1mN of thrust, relatively small level of specific impulse
(4) Plasma microthruster	Low thrust (0.04–1.4 mN) High specific impulse (50–4300 s)	Low-volume, low-cost, low-weight, and high reliability	Needs a higher operating voltage
(5) Colloid microthruster	Low thrust (< 20 μN) High specific impulse (500–1300 s)	Relatively large specific impulse range, high thrust accuracy, and low thrust noise	Needs a higher working voltage
(6) Electrospray microthruster	Low thrust (30–65 μN) High specific impulse (>1000 s)	A high specific impulse with low flow rate, high efficiency and operational flexibility	Too low thrust, in the micro Newton-scale
(7) Free molecule micro–resistojet (FMMR)	Low thrust (< 35 μN) High specific impulse (4000–8000 s)	Low thrust noise, high thrust accuracy, and repeatability.	Relatively bigger volume, short lifetimes, require a large power
(8) Cold gas microthruster (CGM)	Low thrust (0.8–2.24 mN) Low specific impulse (< 50 s)	Simple structure, reliable, low energy consumption, and easy to be miniaturized	Needs high pressure gas storage tank, large volume and weight, difficult to prevent leakage

**Table 9:** A comparison of four kinds of mainly used bonding methods.

Bonding Methods	Advantages	Disadvantages
Adhesive bonding	Simple operation, convenient to fabricated, low cost, no limit to the use of the material.	The sealing effect is poor, and the epoxy resin adhesive used must be high-temperature resistant.
Anodic bonding	No intermediate layer, good airtightness and long-term stability, high degree of combination.	Stress is easily generated after bonding.
Eutectic bonding	No need for high temperature (only about 400 °C) and high voltage, the required for the surface smoothness of silicon is also not high.	A strong electrostatic field is required, which may cause deformation of the fragile structure.
LTCC/HTCC process bonding	Complete bonding in sintering of the ceramic, has good airtightness.	Has limited in use condition of material.
